# Early cerebrospinal fluid elevations of pTau-217 in severe traumatic brain injury subjects

**DOI:** 10.3389/fneur.2025.1632679

**Published:** 2025-07-30

**Authors:** Hamad Yadikar, Firas H. Kobeissy, Claudia Robertson, Spyridoula Tsetsou, John B. Williamson, Damon G. Lamb, Amy K. Wagner, Todd Kibaugh, Shih-Han Kao, Zhifeng Kou, Robert D. Welch, Jose-Miguel Yamal, Luis Leon-Novelo, Richard Rubenstein, Kevin K. W. Wang

**Affiliations:** ^1^Department of Biological Sciences, Faculty of Science, Kuwait University, Kuwait City, Kuwait; ^2^Center for Neurotrauma, Multiomics & Biomarkers, Department of Neurobiology, Neuroscience Institute, Morehouse School of Medicine, Atlanta, GA, United States; ^3^Department of Biochemistry and Molecular Genetics, Faculty of Medicine, American University of Beirut, Beirut, Lebanon; ^4^Department of Emergency Medicine, University of Florida, Gainesville, FL, United States; ^5^Brain Rehabilitation Research Center, Malcom Randall VA Medical Center, Gainesville, FL, United States; ^6^Center for Visual & Neurocognitive Rehabilitation (CVNR), Atlanta VA Health Care System, Decatur, GA, United States; ^7^Department of Neurosurgery, Baylor College of Medicine, Houston, TX, United States; ^8^Department of Psychiatry, College of Medicine, University of Florida, Gainesville, FL, United States; ^9^Department of Physical Medicine & Rehabilitation, University of Pittsburgh, Pittsburgh, PA, United States; ^10^Safar Center for Resuscitation Research, University of Pittsburgh, Pittsburgh, PA, United States; ^11^Department of Neuroscience, University of Pittsburgh, Pittsburgh, PA, United States; ^12^Center for Neuroscience, University of Pittsburgh, Pittsburgh, PA, United States; ^13^Clinical and Translational Science Institute, University of Pittsburgh, Pittsburgh, PA, United States; ^14^Resuscitation Science Center of Emphasis, Department of Anesthesiology and Critical Care Medicine, The Children's Hospital of Philadelphia, School of Medicine at the University of Pennsylvania, Philadelphia, PA, United States; ^15^College of Engineering, School of Medicine, Wayne State University, Detroit, MI, United States; ^16^HUH-MR Research/Radiology, Detroit, MI, United States; ^17^Department of Emergency Medicine, Wayne State University School of Medicine, Detroit, MI, United States; ^18^Department of Biostatistics and Data Science, School of Public Health, The University of Texas Health Science Center at Houston, Houston, TX, United States; ^19^Department of Neurology, SUNY Downstate Health Sciences University, Brooklyn, NY, United States; ^20^Department of Physiology/Pharmacology, SUNY Downstate Health Sciences University, Brooklyn, NY, United States; ^21^Department of Chemistry, Chemistry Laboratory Building, University of Florida, Gainesville, FL, United States

**Keywords:** traumatic brain injury, CSF biomarkers, pTau-217, neurotrauma prognostics, diagnostic biomarkers

## Abstract

**Introduction:**

Tauopathies, including Alzheimer’s disease (AD), feature abnormal accumulations of hyperphosphorylated Tau protein; however, their biomarker potential in traumatic brain injury (TBI) is not well-defined. This study investigated whether cerebrospinal fluid (CSF) phosphorylated Tau at threonine-217 (pTau-217) could serve as an early biomarker for severe TBI (sTBI).

**Methods:**

CSF samples from 26 sTBI patients, collected between 6 and 240 h post-injury, and 19 healthy controls were analyzed using an optimized direct enzyme-linked immunosorbent assay (ELISA; sensitivity <4.7 pg/mL) for pTau-217 detection, complemented by Western blot validation. Temporal analysis, ROC curves, and trajectory clustering were used for interpretation.

**Results:**

CSF pTau-217 levels were significantly elevated in sTBI patients at 6, 12, 18, 24, and 48 h post-injury compared to controls (*p* < 0.05–*p* < 0.001), peaking around 18 h (~65 ng/mL) before declining to near-control levels by 120 h. ROC analyses showed AUC of 0.78 (6–12 h) and 0.83 (24–48 h). Clustering identified a subgroup with sustained high pTau-217, associated with diffuse axonal injury and worse 6-month outcomes. A significant inverse correlation was observed between CSF pTau-217 at 24–48 h and GOSE (ρ = –0.67, *p* < 0.01).

**Discussion:**

These findings indicate that CSF pTau-217 is a sensitive and early biomarker of acute tau pathology in sTBI. Its diagnostic performance and association with axonal injury and outcome support its utility, though longitudinal validation in larger cohorts is required to confirm clinical relevance.

## Introduction

Traumatic brain injury (TBI) is a primary global public health concern characterized by varying severity and clinical outcomes, demanding reliable early biomarkers for diagnosis and prognosis (1–3). Older adults, mainly those aged 75 and above, experience the highest rates of TBI-related deaths and hospitalizations (4). Males are disproportionately affected, with rates of deaths and hospitalizations significantly higher than those for females. Racial and ethnic disparities are also evident, with American Indian/Alaska Native populations experiencing the highest rates of TBI-related deaths (5, 6). TBI is a mechanical injury traumatically induced by an external force that leads to physiological disruption of brain function, indicated by specific symptoms that immediately follow the event or are delayed (7). These forces include direct impacts, penetrating injuries, or blast effects (7). In the clinical evaluation of unconscious states, loss or alteration of consciousness and altered mental states post-injury are pivotal in differentiating TBI from non-traumatic unconscious episodes (8, 9). These symptoms indicate TBI when they occur in head trauma, as opposed to unconsciousness arising from non-traumatic causes, such as metabolic imbalances or cardiovascular events. The resulting neurological deficits may be temporary or permanent, such as direct memory loss for the events before or after the injury, and intracranial lesions (10). TBIs are classified into mild, moderate, or severe categories by clinical characteristics (11).

Tau protein stabilizes neuronal microtubules, and its pathological hyperphosphorylation plays a crucial role in TBI-related neurodegeneration (12–14). Phosphorylated Tau (pTau) and neurofibrillary tangles (NFTs) can be observed as early as 6 h after a moderate-to-severe TBI (msTBI) occurrence (15, 16). Increased NFT levels have been noticed in roughly one-third of post-mortem people who survived a msTBI, which suggests the link between tau aggregation and a single TBI (17).

Abnormal metabolism of amyloid-β (Aβ) and tau causes a buildup of extracellular plaques composed of misfolded Aβ and intraneuronal NFT made of pTau protein in Alzheimer’s disease (AD) (18). Significant progress in AD diagnosis has been made in the last few years, such as developing tests for site-specific pTau biomarkers (pTau-181 and pTau-217) to differentiate AD from non-AD in brain tissues, CSF, and plasma samples (19). Cerebrospinal fluid (CSF) pTau-181 is increasingly recognized as a valuable biomarker for AD, showing promise in diagnostic specificity and detecting AD pathology (20–23). However, it was found that CSF pTau-217 more accurately represents tau pathology associated with AD (24). One of the most concerning sequelae of TBI is the potential for an increased risk of AD (25, 26). The relationship between TBI and AD has been the subject of extensive research, with studies suggesting a complex interplay between the severity of the initial brain injury and the subsequent risk of developing AD. The severity of TBI is typically classified based on clinical criteria, such as the duration of loss of consciousness, the depth of post-traumatic amnesia, and neurological deficits. These classifications range from mild to severe and indicate the injury’s immediate impact on brain function. However, the repercussions of TBI extend beyond these immediate effects, with evidence suggesting that the severity of the injury may have a proportional relationship with the risk of later-life cognitive impairments, including AD (27). Epidemiological studies show that msTBIs increase the risk of AD compared to milder TBIs or no brain injury (28). A study stratified TBI severity indicated that ‘probable’ and ‘possible’ TBIs increased the incidence of AD and related dementias (ADRD), while ‘definite’ TBI did not (25). This shows that ADRD risk may not be uniform across various TBI severities, emphasizing the need for a better understanding of how brain damage affects neurodegenerative diseases. Research suggests that TBI severity is linked to AD risk through neuroinflammatory reactions, amyloid-beta (Aβ) peptide accumulation, and tau protein hyperphosphorylation, which are common markers of AD pathology (26). Extreme TBIs may accelerate AD incidence and progression by worsening these mechanisms. pTau-217 is a promising AD biomarker, especially after TBI. High plasma pTau-217 levels may indicate acute tau pathology alterations post-TBI, predicting AD risk in TBI patients (29). This connection provides insights into the neuropathophysiological processes that link TBI severity to later-life cognitive deficits, such as AD (30).

Identifying biomarkers for developing non-invasive or minimally invasive and inexpensive monitoring tests in patients with TBI is an urgent and unmet need. CSF pTau directly indicates the pTau state in the brain (30–32). So far, the patient-reported symptoms obtained by the Rivermead Post-concussion Symptoms Questionnaire, Post-Concussion Symptom Scale (PCSS), and the Sport-related Concussion Assessment Tool 5th Edition (SCAT5) are the basis for the most valid clinical mild TBI (mTBI) assessments (33, 34, 96). These highly subjective tests cause conflict in estimating injury severity (35).

Current clinical assessments and neuroimaging have limitations in objectively capturing early and subtle biochemical changes in TBI patients, highlighting the critical need for sensitive and specific biomarkers (36–39). Given the importance of early tau pathology post-injury, this study aims to evaluate CSF pTau-217 as a potential biomarker for the early diagnosis of severe TBI. We hypothesize that CSF pTau-217 is significantly elevated in the early post-injury phase in patients with severe TBI, providing a reliable diagnostic tool for acute neuronal injury.

## Materials and methods

Control and TBI CSF subjects: Healthy control CSF samples (*n* = 19, 79.0% male, average age ± SD: 42.4 ± 17.6 years) were acquired in 1 mL aliquots via lumbar puncture (BioIVT, Westbury, NY, United States). BioIVT is a commercial biobank that rigorously screens donors, explicitly excluding individuals with documented neurological, cardiovascular, renal, metabolic (including diabetes and obesity), autoimmune diseases, or conditions known to affect blood–brain barrier permeability. CSF samples from patients with sTBI [*n* = 26, male 92.3%, average age (±SD) 30.7 ± 11.1 years] were obtained frozen from archived collections at Baylor College of Medicine (BCM) (40) (Table 1). A multifactorial randomized clinical trial of erythropoietin vs. placebo and blood transfusion thresholds in sTBI patients was the original study that prompted CSF collection. The entire protocol for this study is detailed in the research paper (40). The BCM IRB approved the study protocol, and a qualified hospital employee performed the procedures in accordance with the hospital’s standard operating procedures.

**Table 1:** Demographic and clinical characteristics of traumatic brain injury patients compared to healthy controls.

Demographic characteristics	TBI subjects	Healthy control subjects	*p*-value
Number of Subjects	26	19	
Age (years), Mean ± SD	30.7 ± 11.1	42.4 ± 17.6	0.017
Sex, *n* (%)			
Male	24 (92.3%)	15 (79.0%)	0.377
Female	2 (7.7%)	4 (21.0%)	
Race/Ethnicity, *n* (%)			
White	4 (15.4%)	4 (21.0%)	0.651
Black	4 (15.4%)	4 (21.0%)	
Hispanic	18 (69.2%)	10 (58.0%)	
Clinical injury metrics			
Admission GCS score, median (IQR), *n* (%)			
3–5	8 (30.8%)		
6–8	13 (50.0%)		
9–11	5 (19.2%)		
Admission mGCS, *n* (%)			
1–3	10 (38.5%)		
4–5	16 (61.5%)		
Number reactive pupils, *n* (%)			
0–1	16 (61.5%)		
2	10 (38.5%)		
Marshall CT score, *n* (%)			
Diffuse Injury II (D2)	14 (53.8%)		
Diffuse Injury III (D3)	5 (19.2%)		
EM	7 (26.9%)		
Injury Severity Score	29.8 ± 7.3		
DRS	9.6 ± 8.6		
6 Month GOS-E, *n* (%)			
Favorable	8 (30.8%)		
Unfavorable	17 (65.4%)		
Lost to follow-up	1 (3.8%)		

TBI, traumatic brain injury; HC, healthy control; GCS, Glasgow Coma Scale; ISS, injury severity score; GOS-E, Glasgow Outcome Scale-Extended. The demographic characteristics, including age, gender distribution, and race/ethnicity, are presented as numbers (percentages) for categorical data and as means (standard deviations) for continuous variables. Clinical parameters, such as GCS scores, ISS, and GOS-E outcomes, are reported as the median (interquartile range). Statistical significance (*p*-value) is denoted by ‘n.s.’ for non-significant, with a *p*-value > 0.05 indicating no statistical difference between TBI and HC groups. The injury characteristics reflect the initial assessment upon admission and subsequent evaluations of injury severity and patient outcomes.

### Sample cohort specifications

Severe traumatic brain injury (sTBI) was defined explicitly using the Glasgow Coma Scale motor component score ≤5. This approach aligns with the original inclusion criteria of Robertson et al. (41), from whom our cohort was derived. It is consistent with standard definitions widely utilized across recent TBI studies (42). The motor component of the Glasgow Coma Scale (GCS) is recognized as the most reliable and objective indicator for evaluating acute neurological impairment, especially when verbal or eye components are compromised by sedation or intubation (43, 44). It provides comparable or superior prognostic value relative to the full GCS score (45–47).

### Handling of GCS data in intubated patients

The GCS score was employed as a standardized measure of injury severity, with specific adjustments made for intubated patients at admission. A verbal score of 1 was assigned in these cases, consistent with the methodology outlined in the original clinical trial (41). This adjustment was applied to a small proportion of the cohort to address the inherent limitations of verbal GCS scoring in intubated patients while consistently calculating the total GCS score. To further ensure transparency and rigor, intubated patients were identified and designated clearly in analyses, including scatter plots and statistical assessments. This designation allows for nuanced interpretation of the data while maintaining consistency with established clinical definitions.

### CSF sample collection protocol

Cerebrospinal fluid samples from sTBI patients were collected exclusively from external ventricular drains placed as standard care for intracranial pressure management, not via lumbar puncture. Healthy control CSF samples, however, were obtained via lumbar puncture solely for baseline comparison purposes. CSF samples were collected for up to 10 days or until an intraventriculostomy was no longer required clinically. sTBI patient CSF samples were collected at the following time points: 6, 12, 24, 48, 72, 96, 120, 168, 192, and 240 h post-injury. CSF were sampled from the buretrol of the CSF drainage system with a total collection time not exceeding 1 h and were diverted to 15-mL conical polypropylene centrifuge tubes (BD Falcon, San Jose, CA, United States). At room temperature, CSF samples were centrifuged at low speed using a tabletop centrifuge (4,000 × *g*) for 5–7 min to remove loose cells and debris. One mL aliquot of the debris-free CSF (supernatant) was pipetted into 2 mL cryogenic tubes, snap-frozen, and stored at −80°C.

### Human SH-SY5Y neuronal cell line

Cells were obtained from ATCC (Catalog# CRL-2266) and maintained in Dulbecco’s modified Eagle medium (DMEM, Gibco, Gaithersburg, MD, United States, cat. no. 31885-049), enriched with 10% Fetal Bovine Serum (FBS, Gibco, Catalog# 10437-028), 1% L-glutamine (Gibco, cat. no. 25030024), 1% non-essential amino acids (Sigma-Aldrich, Darmstadt, Germany, cat. no. M7145) and 1% Penicillin–Streptomycin solution (Gibco, Catalog# 15140-122). Cells were incubated at 37°C in a humidified 5% CO_2_-containing atmosphere. The medium was refreshed every 3 days until the desired confluency was achieved. SH-SY5Y cells grown to 60–90% confluency were used. For phosphatase inhibition, okadaic acid (Sigma-Aldrich, Catalog# O8010) was prepared as a 1 mM stock in DMSO and diluted in the culture medium to achieve final concentrations of 100 nM and 1 μM. The cells were treated for 24 h, ensuring the DMSO concentration did not surpass 0.1% of the total volume. Following treatment, cells were washed with Phosphate-Buffered Saline (PBS, Gibco, Catalog# 10010-023), detached using Trypsin–EDTA solution (Gibco, Catalog# 25300-054), neutralized with complete medium, and pelleted by centrifugation.

After the respective treatments, the culture medium was aspirated from each well and discarded. The cells were rinsed with ice-cold Phosphate-Buffered Saline (PBS) to remove residual medium and detached cellular debris. The PBS was removed, and each well was treated with 100 μL of ice-cold Triton-X lysis buffer per well. The Triton-X lysis buffer composition included 1 mM DTT (Dithiothreitol), 1% (v/v) of phosphatase inhibitors cocktail (Sigma-Aldrich, Catalog# P0044), 1% (v/v) Mini-Complete protease inhibitor cocktail (Roche Biochemicals, Catalog# 11836153001), and 1% (v/v) Triton X-100 (Sigma-Aldrich, Catalog# T8787), prepared in PBS. Using a cell scraper, the cells were gently detached from the surface of the culture wells, and the lysate was collected into pre-cooled 1.5 mL Eppendorf tubes. The tubes were incubated on a rotator for 90 min at 4°C to ensure thorough cell lysis. After incubation, the lysates were centrifuged at 15,000 × g for 15 min at 4°C. The resultant supernatant, containing the solubilized proteins, was transferred to new tubes, being cautious to avoid the insoluble pellet. Following the manufacturer’s protocol, the cell lysate was quantified for protein concentration using the BCA Protein Assay (Thermo Fisher Scientific, Catalog #23225). The lysates were used immediately for downstream applications or aliquoted and stored at −80°C for long-term storage. This method ensured the preservation of protein phosphorylation states and minimized protein degradation, allowing for accurate downstream biochemical analysis.

### Transgenic htau mouse brain lysate

We used the human tau (Htau) transgenic mouse model for our study (48). Animal experiments were conducted following the NIH’s Guide for the Care and Use of Laboratory Animals and approved by the Institutional Animal Care and Use Committee (IACUC) at the University of Florida (approval number: 202110180, approval date: 18 October 2021). Three-month-old htau transgenic mice (*n* = 10; 5 males and 5 females) expressing all human tau isoforms under tau promoter control, and age-matched wild-type control littermates (*n* = 10; 5 males and 5 females) were used to assess the specificity of our pTau-217 antibody. Animals were housed individually under standard conditions (22 ± 1°C, 55 ± 5% humidity, 12-h light/dark cycle), provided with ad libitum food, water, and environmental enrichment, and regularly monitored for signs of distress. Animals displaying significant health issues or weight loss (>10% body weight/week) were excluded (none were excluded in this study). Investigators remained blinded to genotype during all analyses. Mice were anesthetized with isoflurane, euthanized via decapitation, and brain regions (cortex and hippocampus) immediately dissected and snap-frozen in liquid nitrogen.

The tissues were ground into a fine powder using a mortar and pestle on dry ice, with liquid nitrogen used for cooling. Brain powder was placed into microcentrifuge tubes. We lysed the brain powder with a 1% Triton X-100 lysis buffer that included 20 mM Tris HCl (pH 7.0), 5 mM EDTA, and 1 mM DTT, all dissolved in LC–MS grade water. The lysates were mixed at low speed on a tube revolver at 4°C for 120 min, then centrifuged at 10,000 × g for 15 min at the same temperature. The clear supernatant was collected into new tubes. Protein concentrations were measured using a bicinchoninic acid protein assay. The lysates were stored at −80°C until needed. The procedures involving animals were conducted in accordance with the NIH’s Guide for the Care and Use of Laboratory Animals and received approval from the University of Florida’s Institutional Animal Care and Use Committee.

### Quantitative immunoblotting

Ten microliters of control and TBI-CSF samples were mixed with eight micromolars of SDS sample buffer [50 mM Tris, pH 6.8, 25 mM DTT, 2.5% SDS, 0.02% bromophenol blue (BPB), and 25% glycerol]. Equal amounts of protein (20 μg) were loaded onto Tris/glycine gels (Invitrogen Life Technologies) and then separated by electrophoresis at 200 V for 60 min. Proteins were transferred to a polyvinylidene difluoride membrane (Invitrogen) using the iBlot Gel Transfer Device (Invitrogen) for 7 min. Following the transfer, the membranes were blocked in 5% non-fat dry milk in Tris-buffered saline with 0.1% Tween 20 (TBST; 20 mM Tris–HCl, 150 mM NaCl, and 0.1% Tween-20, pH 7.5) for 1 h. Polyclonal anti-rabbit pTau-217-specific antibody (Assay Genie, catalog# AEFI00429, Ireland) was incubated with immunoblotting membranes at a dilution of 1: 1000 in 5% milk at 4°C overnight. The following day, the membranes were washed three times with TBST and probed with an alkaline phosphatase-conjugated anti-rabbit IgG antibody (EMD Millipore, MA, United States) at a dilution of 1:5000 in 5% non-fat dry milk for an hour, followed by TBST washing. Immunoreactivity was detected using BCIP/NBT (Kirkegaard & Perry Laboratories, Gaithersburg, MD, United States). Band intensity was quantified by NIH ImageJ v1.7 software.

### Direct ELISA for CSF pTau-217

CSF aliquots (10 μL) were adsorbed to high-binding ELISA plates, blocked, and probed with a phosphorylation-specific anti-pTau-217 antibody followed by HRP-conjugated secondary antibody (see Supplementary Figure S1 for workflow). Signal was developed with TMB, stopped with 2 M H₂SO₄, and read at 450 nm. Purified recombinant Tau-441 (non-phosphorylated) and dual-kinase–phosphorylated Tau-441 standards (0–1 μg mL^−1^) generated a 10-point standard curve (*R*^2^ = 0.84). Total-Tau and rabbit-IgG isotype controls verified specificity; assay background was <5% of the lowest standard. All samples were run in duplicate; intra-assay CV < 8%, inter-assay CV < 10%. A detailed description of the assay optimization, including antibody titration curves and standard curve development, is provided in the Supplementary File 1.

### Statistical analysis

**Figure 1:**
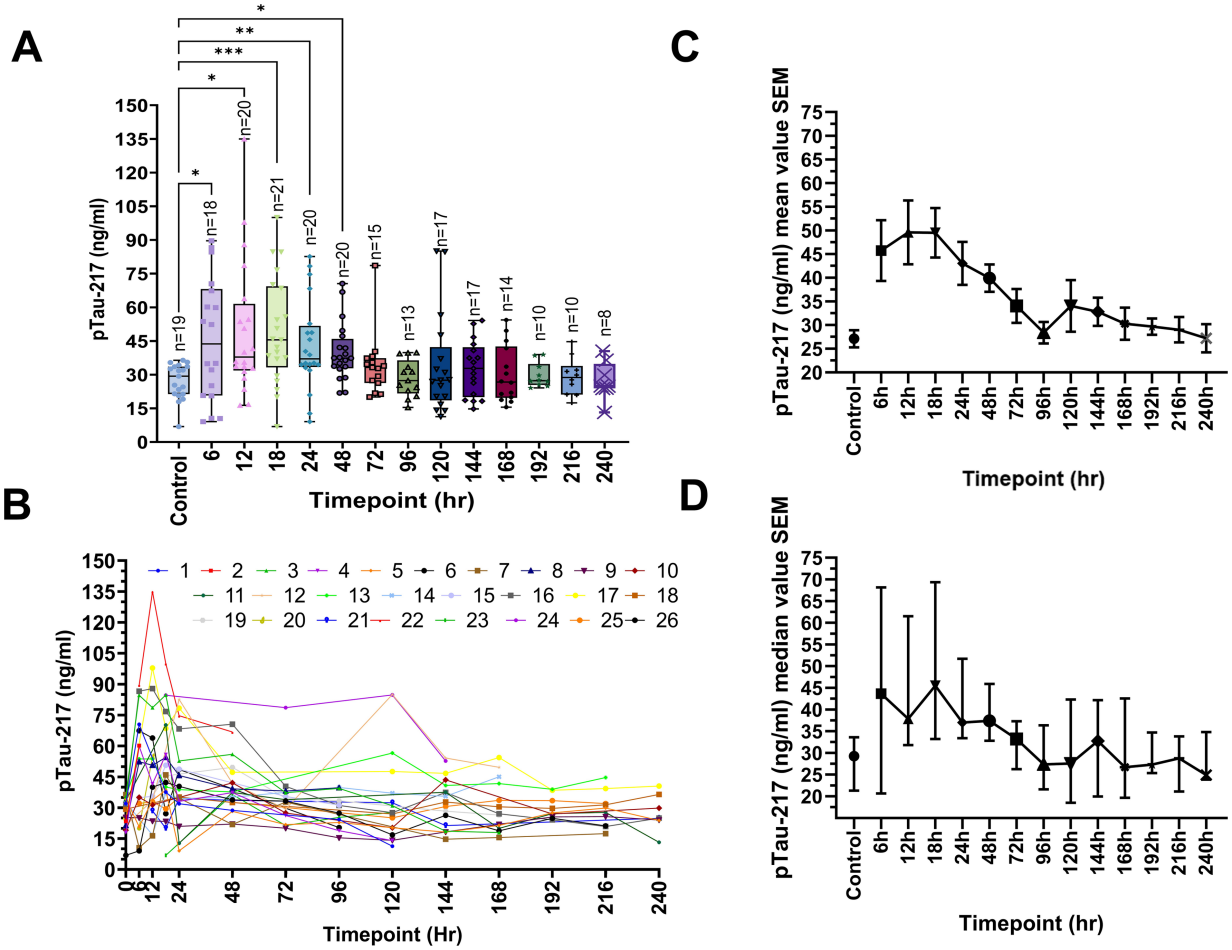
Temporal evolution of CSF pTau-217 levels in severe TBI. **(A)** This box plot illustrates the temporal profile of pTau-217 concentrations (ng/ml) in the CSF of 26 severe TBI patients over a range of post-injury time points (6–240 h). Each box represents the interquartile range (25th to 75th percentile), with whiskers extending to the minimum and maximum values. The horizontal line within each box indicates the median concentration. Statistically significant differences in pTau-217 levels between controls and TBI patients are marked by asterisks (*p < 0.05, **p < 0.01, ***p < 0.001). **(B)** A spaghetti-line graph presents the individual pTau-217 concentrations (ng/ml) for each patient at multiple time points post-injury, highlighting the variability across the cohort. Each line corresponds to a unique patient, capturing the dynamics of pTau-217 over time. **(C)** This line graph shows the mean pTau-217 concentrations (ng/ml) in CSF across all patients at each time point, with error bars representing the standard error of the mean (SEM). **(D)** A line graph displays the median pTau-217 concentrations (ng/ml) at various time points, along with the SEM. Data were analyzed using one-way ANOVA with Bonferroni correction, followed by *post hoc* pairwise comparisons to assess the significance of changes in pTau-217 levels over time. TBI, traumatic brain injury; CSF, cerebrospinal fluid; OD, optical density; SEM, standard error of the mean; ANOVA, analysis of variance.

Data analyses were performed using GraphPad Prism (version 10.2.1) and Python (version 3.10.4). Descriptive statistics for demographic and clinical data were reported as mean ± standard deviation (SD) to reflect sample variability (Table 1). In contrast, experimental data comparing group trends were reported as mean ± standard error of the mean (SEM) (Figure 1). Normality was assessed using the Shapiro–Wilk test to determine the appropriate statistical tests. For normally distributed continuous variables, parametric tests were applied: unpaired t-tests were used to compare two independent groups, and one-way analysis of variance (ANOVA) followed by Tukey’s multiple comparisons test was applied for more than two groups. For continuous data not normally distributed, non-parametric tests were employed: Mann–Whitney *U* tests for two-group comparisons and Kruskal–Wallis tests for comparisons involving more than two groups. Categorical data (such as gender, race/ethnicity) were analyzed using Fisher’s exact test.

Mixed-effects models were implemented to analyze longitudinal repeated measures, incorporating patient-specific random intercepts to account for individual variability. Fixed effects included time post-injury, clinical treatment conditions, and demographic factors (age, gender). To validate model assumptions, residuals from mixed-effects models were visually and statistically inspected for normality, homoscedasticity, linearity, and independence. Correlation analyses were conducted using Pearson correlation and linear regression to examine relationships between admission GCS scores (independent variable) and CSF pTau-217 levels at 24–48 h post-injury (dependent variable). Patients missing GCS or pTau-217 data at the relevant time points were excluded from correlation analyses. Statistical significance for correlation was set at *p* < 0.05.

Receiver operating characteristic (ROC) analyses were conducted to evaluate the diagnostic accuracy of pTau-217 in distinguishing TBI patients from controls. Area under the curve (AUC) values assessed biomarker discriminatory performance, categorized as excellent (0.8–1.0), adequate (0.7–0.8), or poor (0.5–0.7). Optimal biomarker cutoffs were established using Youden’s Index. Levels of statistical significance were indicated as follows: *p < 0.05, **p < 0.01, ***p < 0.001, and ****p < 0.0001. For heatmap visualization, pTau-217 data measured across multiple post-injury time points were normalized using *Z*-scores to emphasize relative intra-patient biomarker dynamics and minimize baseline variability between patients. Hierarchical clustering grouped patients based on similarity in temporal pTau-217 patterns, utilizing Euclidean distance and Ward’s linkage method. Missing values were not imputed and were represented as gray cells in the heatmap. Heatmaps and clustering analyses were conducted using Python’s Seaborn (version 0.12.2) and SciPy (version 1.10.1) libraries.

## Results

Our study utilized a pTau-217 antibody to examine CSF samples from individuals with sTBI, as this biofluid compartment may reflect acute brain changes. The capability of the assay to detect and measure pTau-217, a phosphorylated tau variant relevant to AD and potentially TBI, is demonstrated (Supplementary Figure S1). The human brain’s longest isoform (2N4R), along with its domains, regions, phosphorylation sites, and antibody recognition sites, is illustrated in Supplementary Figure S2. In healthy brains, tau proteins are minimally phosphorylated, and the level of phosphorylation increases in neurodegenerative disorders such as AD or frontotemporal lobar degeneration (19, 49). The pTau-217 epitope resides within the projection domain, which is more accessible to antibodies than other buried regions of the tau protein. These features highlight the utility of pTau-217 as a potential biomarker for neurotrauma and related conditions (50–52).

### Sample characteristics

Our study included 45 participants, divided into patients with sTBI (*n* = 26) and healthy controls (*n* = 19) (Table 1). The sTBI group had a mean age of 30.7 ± 11.1 years, which was significantly younger than that of the controls [42.4 ± 17.6 years; Welch’s *t*-test: *t*(28) = 2.55, *p* = 0.017]. However, linear regression analysis revealed no significant relationship between age and pTau-217 levels at any measured time point (all *p* > 0.05; Supplementary Figure S4), suggesting that age had a minimal influence on biomarker concentrations in our cohort. To account for the baseline age difference between groups, age was included as a covariate in all relevant statistical models, including the mixed-effects model for longitudinal analysis. The gender distribution did not differ significantly between groups (Fisher’s exact test, *p* = 0.377; Table 1). Participants showed diverse ethnicities: the TBI group was predominantly Hispanic (69.2%), with 15.4% Black and 15.4% White, which was not significantly different from the control group’s distribution [Hispanic 58%, Black 21%, White 21%; *χ*^2^(2) = 0.86, *p* = 0.65].

Injury characteristics (for TBI patients) indicated a range within severe TBI: admission GCS scores of 3–5 in 30.8%, 6–8 in 50.0%, and 9–11 in 19.2%. The median Injury Severity Score (ISS) was 29.8 (reflecting very severe overall trauma). Analysis of pTau-217 levels by racial/ethnic groups (Supplementary Figure S5) showed no significant differences at most time points (*p* > 0.05 for group effect). However, at some later time points (96 h, 192 h, 216 h, 240 h), significant differences were observed (*p* < 0.05), with Black patients exhibiting higher pTau-217 levels. This finding highlights the importance of demographic stratification in exploratory biomarker analyses. Glasgow Outcome Scale-Extended (GOS-E) scores at 6 months differed among TBI subjects: 30.8% had favorable outcomes (good recovery), 65.4% had unfavorable outcomes (severe disability/vegetative/death), and 3.8% were lost to follow-up; by definition, all controls had favorable outcomes.

### Direct ELISA assay of CSF pTau-217

The schematic outlines the key steps of the ELISA protocol used for quantifying pTau-217 in CSF samples from TBI and control subjects (Supplementary Figure S1). The specific locus of the pTau-217 epitope within the human tau-441 protein resides within the proline-rich domain (Supplementary Figure S2). This region is a phosphorylation hotspot containing multiple post-translationally modified sites in various neurodegenerative conditions (53, 54). The figure presents the pTau-231, pTau-202/205, and pTau-181 epitopes alongside the site recognized by the anti-pTau-217 antibody. The previously established epitopes pTau-202 (21B2) and pTau-396/404 (4E7-1), as well as the new pTau-217 site, are comparatively assessed.

**Figure 2:**
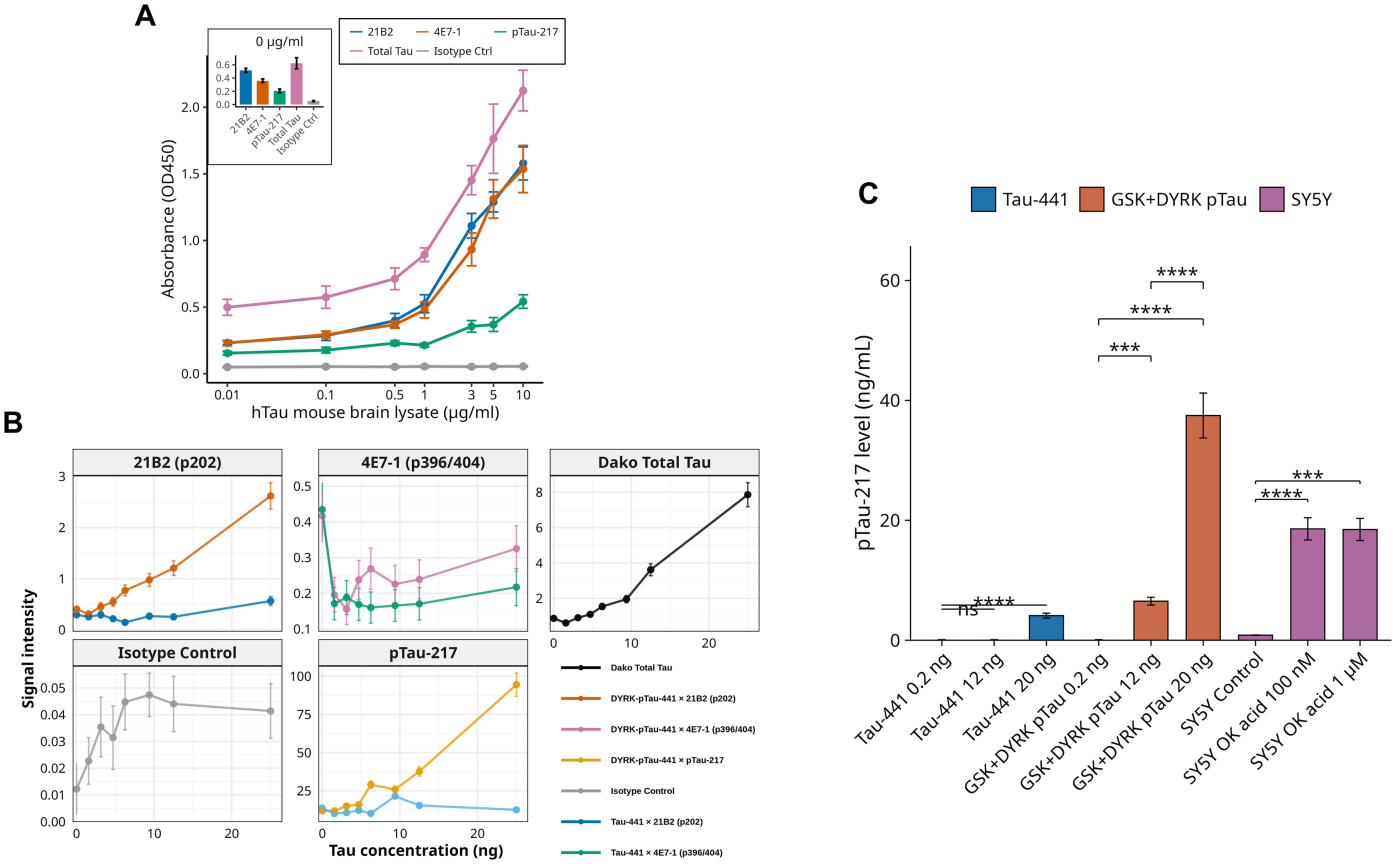
Characterization of prototype pTau-217 assay specificity for phosphorylated Tau. **(A)** Dose-dependent ELISA analysis comparing binding of Tau antibodies (21B2, 4E7-1, pTau-217) to human Tau (hTau) mouse brain lysate across a concentration range (0.01–10 μg/mL). Total Tau antibody (Dako, Cat. #A0024) was a positive control, while a rabbit IgG isotype antibody (Cell Signaling Technology, Cat. #3900) was a negative control. Inset represents antibody binding signals at 0 μg/mL lysate concentration. Data represent means ± SEM of three independent replicates measured as absorbance at 450 nm (OD450). **(B)** Antibody binding profiles against recombinant Tau-441 (non-phosphorylated vs. DYRK1A-phosphorylated). Signal intensities of phosphorylation-specific antibodies (21B2 targeting pSer202, 4E7-1 targeting pSer396/404, and pTau-217 targeting pThr217) were evaluated at increasing Tau concentrations (0–25 ng). The pan-specific Dako total Tau antibody served as a reference control. Antibody 21B2 selectively detects DYRK1A-phosphorylated Tau at Ser202, 4E7-1 moderately favors phosphorylated Tau at Ser396/404, and pTau-217 antibody exhibits robust and selective recognition of Thr217-phosphorylated Tau (*p* < 0.001). Dako total Tau antibody detected phosphorylated and non-phosphorylated Tau forms, showing slightly increased affinity toward phosphorylated Tau (*p* < 0.05). Isotype control showed negligible binding. Data represent mean ± SEM (*n* = 3). **(C)** Quantifying Tau phosphorylation at Thr217 under various conditions using the prototype pTau-217-specific ELISA. Recombinant Tau-441 (unphosphorylated) and dual-kinase (GSK-3β + DYRK1A)-phosphorylated Tau-441 were measured at 0.2 ng, 12 ng, and 20 ng. Additionally, human neuroblastoma SY5Y cells treated with okadaic acid (OK acid; 100 nM or 1 μM, 24 h) or untreated (control) were analyzed at 20 μg total protein. pTau-217 concentrations (ng/mL) were calculated from OD450 using a third-degree polynomial fit (*R*^2^ = 0.843). Dual-kinase phosphorylated Tau showed significantly enhanced phosphorylation at Thr217 compared to unphosphorylated Tau-441 at 20 ng (**p < 0.001). OK acid induced a dose-dependent increase in pTau-217 levels in SY5Y cells, reaching levels comparable to dual-kinase phosphorylated Tau at 20 ng. Statistical significance assessed by two-tailed Student’s *t*-test: *p < 0.05, **p < 0.01, ***p < 0.001, ***p < 0.0001. Data represent mean ± SEM (*n* = 5). Results confirm Thr217 phosphorylation enhancement by combined GSK-3β and DYRK1A activity, a modification inducible pharmacologically in neuronal cells via phosphatase inhibition.

Antibody responses were quantified across increasing concentrations of hTau mouse brain lysate (0.01–10 μg/mL) (Figure 2A). Antibodies 21B2 (targeting pSer202) and 4E7-1 (targeting pSer396/404) displayed clear dose-dependent binding. Notably, the pTau-217 antibody demonstrated a potent and selective dose–response profile specific for Thr217-phosphorylated Tau. Total Tau antibody (Dako, Cat. #A0024) served as a positive control, confirming the general presence of Tau protein. In contrast, a rabbit IgG isotype antibody (Cell Signaling Technology, Cat. #3900) showed minimal background binding, verifying assay specificity. Antibody concentration optimization (Supplementary Figure S3) demonstrated that anti-pTau-217 antibody concentrations (0.02–10 μg/mL) significantly influenced assay sensitivity. Higher antibody concentrations improved absorbance signals, and pTau-217 concentrations and signal-to-noise ratios were calculated. Optimal detection conditions were established at 10.00 μg/mL (1: 100 dilution), providing maximum sensitivity, specificity, and assay robustness. Antibody binding specificity was evaluated further by comparing non-phosphorylated recombinant Tau-441 versus Tau phosphorylated by DYRK1A kinase (Figure 2B). Antibody 21B2 shows strong selectivity for DYRK1A-phosphorylated Tau at Ser202, and 4E7-1 displays moderate preferential detection at phosphorylated epitopes (pSer396/404). The pTau-217 antibody significantly differentiated (*p* < 0.001) between phosphorylated and non-phosphorylated Tau, demonstrating assay specificity. Dako total Tau antibody recognizes both forms, albeit with a modest preference for phosphorylated variants (*p* < 0.05). The isotype control consistently shows negligible signals, emphasizing assay specificity. Data represent mean ± SEM (*n* = 3 independent replicates).

Recombinant Tau-441 protein (unphosphorylated) and dual-kinase (GSK-3β + DYRK1A)-phosphorylated Tau-441 were evaluated at concentrations of 0.2 ng, 12 ng, and 20 ng (Figure 2C). Additionally, to assess the physiological relevance and translatability of our assay, we included lysates from human neuroblastoma SH-SY5Y cells treated with the phosphatase inhibitor okadaic acid (OA, 100 nM or 1 μM, 24 h) and untreated control lysates at 20 μg total protein (Figure 2C). Including SH-SY5Y cells provides a widely accepted cellular model for studying tau phosphorylation dynamics *in vitro*, enabling validation of our assay in a biologically relevant context. Similarly, hTau mouse brain lysates (from a transgenic mouse model expressing human Tau) were incorporated to validate assay performance using complex biological samples that closely mimic *in vivo* conditions and human tauopathies. pTau-217 concentrations (in ng/mL) were derived from absorbance values (OD_450_) using a third-degree polynomial standard curve (*R*^2^ = 0.843). At 20 ng, dual-kinase phosphorylation significantly increased pTau-217 levels compared to unphosphorylated Tau-441 (*p* < 0.001). Okadaic acid-treated SY5Y cells exhibited a dose-dependent increase in pTau-217, reaching levels comparable to those of dual-kinase phosphorylated Tau at 20 ng (*p* < 0.001).

**Figure 3:**
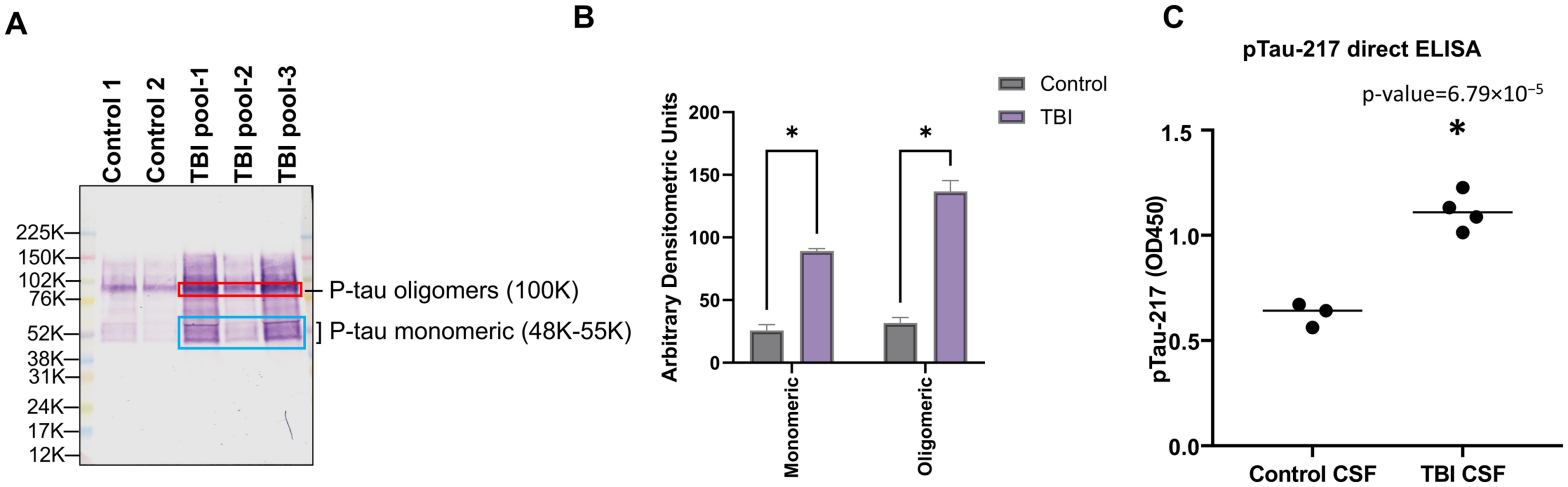
Differential expression of pTau217 forms in CSF following TBI. Panel **(A)** exhibits a Western blot analysis showing tau protein in cerebrospinal fluid (CSF). Two control samples (Control 1, Control 2) and three TBI patient samples (TBI pool-1, TBI pool-2, TBI pool-3) were probed with antibodies specific to phosphorylated tau (pTau-217), captured at 24 h post-trauma. The molecular weights are denoted alongside monomeric pTau species (48K–55K) and oligomeric forms (100K) highlighted by blue and red rectangles, respectively, demonstrating the aggregation state variation between control and TBI samples. Panel **(B)** provides a densitometric analysis of the monomeric (48K–55K) and oligomeric (100K) pTau-217 bands from the Western blot. The bar graph quantifies the band intensities, showing a statistically significant increase in both monomeric and oligomeric pTau-217 levels in the TBI group compared to the control group (*p < 0.05). Panel **(C)** presents a direct ELISA quantification of pTau-217, contrasting the optical density (OD) measurements at 450 nm between control and TBI-derived CSF. The data points for each group indicate individual sample measurements, with the group means depicted by horizontal bars. Compared to controls, a statistically significant increase (*p < 0.05) in pTau-217 levels is observed in TBI samples. HuCSF, human cerebrospinal fluid; OD, optical density; K, KiloDalton.

Western blot analysis reveals a notable increase in monomeric pTau-217 bands (48–55 kDa, blue box) and oligomeric forms (100 kDa, red box) in CSF samples from TBI patients compared to controls, indicating a significant elevation in pTau levels (Figure 3A). The densitometric analysis, as shown in the accompanying bar graph (Figure 3B), quantifies the intensity of these bands, revealing a statistically significant increase in both monomeric and oligomeric pTau forms in the sTBI group compared to the control group (two-sample *t*-test, monomeric: *p* = 1.39 × 10^−5^; oligomeric: *p* = 2.88 × 10^−6^). The unit of measurement, Arbitrary Densitometric Units, reflects the relative amount of protein present, with higher values indicating greater protein concentration. The direct ELISA supports these findings, showing a statistically significant increase in OD_450_ values for TBI-CSF compared to control CSF (two-sample *t*-test, *p* < 0.0001; Figure 3C).

### CSF pTau-217 dynamics and analysis in sTBI

The CSF samples were collected longitudinally (6–240 h post-injury), allowing within-subject analysis. CSF pTau-217 significantly increased during the initial 24 h post-injury, peaking at ~75 ng/mL (median), representing a 3.3-fold rise compared to controls (~25 ng/mL; Figure 1A). Pairwise comparisons between each post-injury time point and controls were performed using the Wilcoxon signed-rank test with Bonferroni correction. Panel A illustrates a box-and-whisker plot of pTau-217 levels measured across post-injury time intervals. Control samples exhibited low baseline pTau-217 levels, clustered tightly around 25 ng/mL. At 6 h post-injury, individual pTau-217 levels showed significant variability, with a statistically significant increase compared to controls (*p* = 0.017, one-way ANOVA with Bonferroni correction). This trend continued at 6–12 h (*p* = 0.0008, one-way ANOVA with Bonferroni correction), with an even greater spread of values, reflecting marked heterogeneity in biomarker expression across patients. The 12- and 18-h time points also showed significant increases (*p* < 0.0001 and *p* < 0.001, respectively, Wilcoxon signed-rank test with Bonferroni correction). At 24–48 h, median pTau-217 levels remained elevated (*p* < 0.01–0.05, Wilcoxon signed-rank test with Bonferroni correction) while individual values began to converge. Beyond 48 h, pTau-217 levels gradually declined, approaching control levels by 96 h. By the final measured interval of 216–240 h, pTau-217 levels were essentially indistinguishable from those of controls, indicating normalization of biomarker expression. It is important to note that the number of patients contributing samples declined over time, as indicated by the “*n*” values in Figure 1A. This variability in sample size could be partly attributed to survival bias, as patients with more severe injuries may have died within the early post-injury period. Additionally, patients contributing samples at later points may represent those with better neurological recovery or lower initial injury severity.

In addition, spaghetti-line time plots illustrate that the temporal profile of individual patient’s CSF pTau-217 levels shows significant variability during the early post-injury hours, with each line representing a single patient’s trajectory over time (Figure 1B). Yet, for most patients, a sharp peak in pTau-217 levels is evident within the first 24 h, followed by a gradual convergence of values across patients (Figure 1B). Figure 1C aggregates these data points into mean pTau-217 concentrations with standard error of the mean (SEM) for each time point. Mean levels peaked significantly within the first 6 h post-injury (mean: ~50 ng/mL) compared to controls (*p* < 0.05, Wilcoxon signed-rank test with Bonferroni correction). They remained elevated during the 6, 12, and 24-h intervals. A marked transition occurred over the 24-h period, with mean pTau-217 levels decreasing to ~30 ng/mL, indicating a shift from acute injury responses to subacute processes. Beyond 48 h, mean pTau-217 levels gradually declined from 120 to 144 h and returned to near baseline levels (~30 ng/mL), indicating the establishment of a new post-injury homeostatic state. The narrowing of SEM observed during this period suggests a reduction in variability among patient responses.

The GCS scores of patients who contributed samples at 24–48 h were analyzed to explore potential biases (Supplementary Figure S6). A borderline significant positive correlation was observed between initial GCS scores and pTau-217 levels at 24–48 h (*p* = 0.054, 𝑟=0.43). This trend suggests that patients with better neurological conditions at admission (higher GCS) might exhibit higher pTau-217 levels at this time, though the evidence is not statistically significant. Therefore, injury severity or survival differences may only partially explain the observed trends in pTau-217 levels. To evaluate whether this reduction could reflect survival bias or differences in initial injury severity, we analyzed GCS scores between early (0–24 h) and late (96–240 h) contributors (Supplementary Figure S7). Patients contributing samples at later time points (96–240 h) displayed a broader distribution of GCS scores, including both low and high severity scores (ranging from 3 to 11). In contrast, early contributors (0–24 h) exhibited a more restricted range concentrated predominantly around moderate-to-higher GCS scores (primarily between 7 and 10).

To further investigate whether the narrowing of the SEM could be explained by differential injury severity, we analyzed the relationship between the ISS and pTau-217 levels across four distinct time intervals (0–6 h, 6–12 h, 12–18 h, and 24–48 h). While weak positive correlations were observed at each interval, none reached statistical significance (*p* > 0.05, Pearson correlation) (Supplementary Figure S7), suggesting that the observed trends in pTau-217 levels are not solely attributable to differences in injury severity. Nonetheless, the shift in cohort composition at later time points likely contributed to reducing variability and stabilizing pTau-217 levels. This transition aligns with the resolution of acute-phase neuropathological processes and a progression toward a subacute or stabilized post-injury phase. For completeness, we also plotted the median CSF pTau-217 ± interquartile range (IQR) (Figure 1D). Again, the median pTau-217 temporal profiles closely align with their mean value counterparts during the early hours. Still, they emphasize persistent heterogeneity within the cohort during the first 24 h (Figure 1D). The gradual stabilization of pTau-217 levels beyond 48 h is evident in both mean and median data, reinforcing the observed trends.

**Figure 4:**
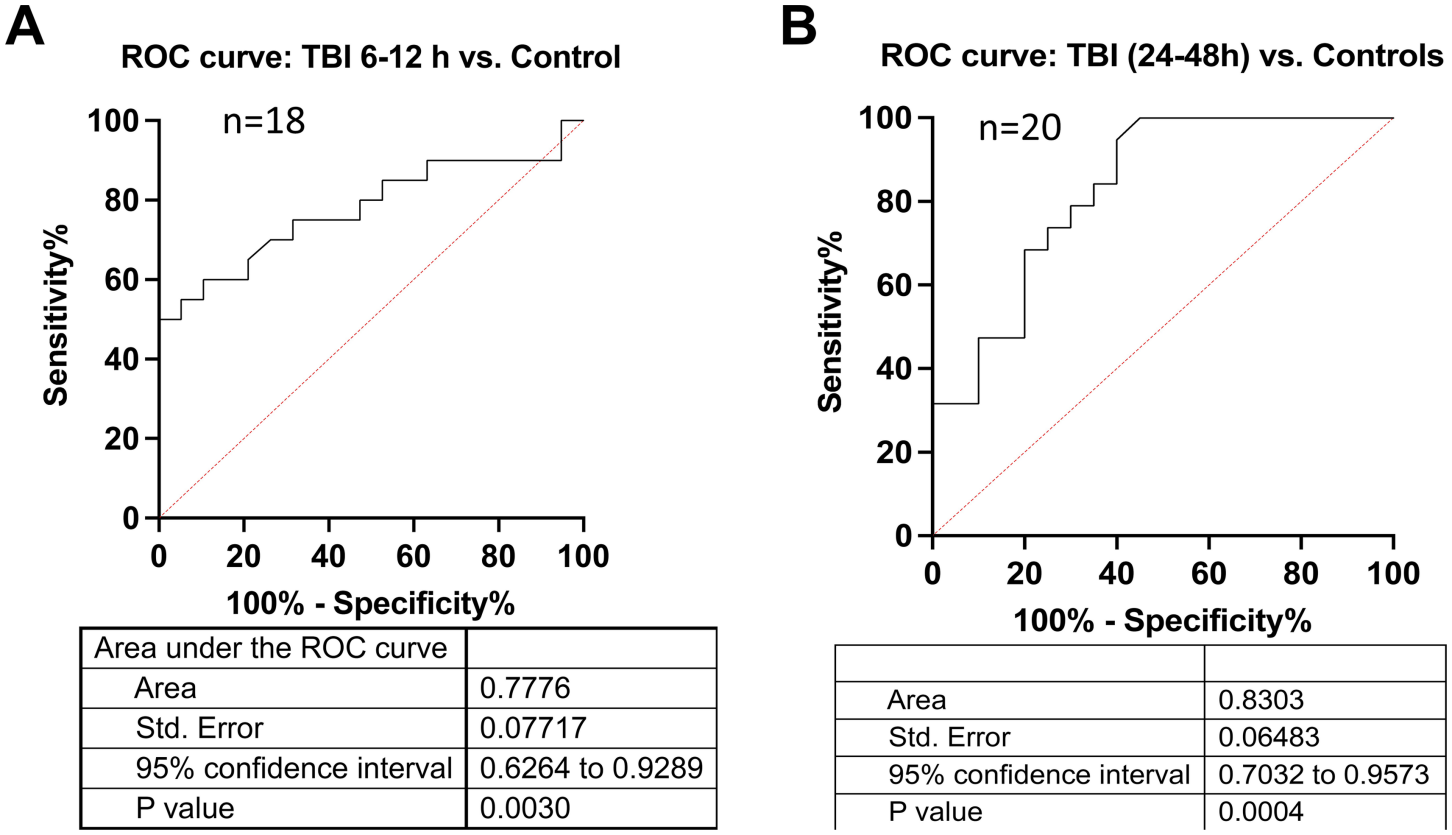
Diagnostic performance of pTau-217 in early TBI detection via ROC analysis. This figure displays Receiver Operating Characteristic (ROC) curves evaluating the diagnostic performance of CSF pTau-217 levels at two critical post-TBI time windows: 6–12 h and 24–48 h, compared to control subjects. **(A)** The ROC curve for the 6–12-h interval post-TBI demonstrates the sensitivity and specificity of pTau-217 as a biomarker to distinguish TBI cases from controls. The AUC of 0.7776 indicates a substantial discriminative ability, with a standard error of 0.07717 and a 95% confidence interval ranging from 0.6264 to 0.9289. The statistical significance of this diagnostic capacity is confirmed by a *p*-value of 0.0030. **(B)** The ROC curve for the 24–48-h interval post-TBI. The standard error is 0.06483, and the 95% confidence interval spans from 0.7032 to 0.9573, with a *p*-value of 0.0004 denoting high statistical significance. ROC, receiver operating characteristic; AUC, area under the curve; CI, confidence interval; TBI, traumatic brain injury; CSF, cerebrospinal fluid.

In the ROC curve analysis, CSF pTau-217 levels demonstrated a significant ability to distinguish between individuals with TBI and controls. At the 6-to 12-h interval, the biomarker exhibited an AUC of 0.78 (95% confidence interval [CI]: 0.63–0.93, *p* = 0.003) (Figure 4A), indicating substantial discriminative power. The maximal specificity of pTau-217 during this time frame reached 100%, meaning it could accurately rule out non-TBI patients while maintaining a sensitivity of approximately 50%, highlighting its robust diagnostic potential in the early phase of the disease. At the 24–48 h interval, pTau-217 continued to demonstrate strong discriminative efficacy, with an AUC of 0.83 (95% CI: 0.70–0.96, *p* < 0.001) (Figure 4B). Although the numerical AUC value increased slightly at the later time point, the broad and overlapping confidence intervals indicate that this difference does not reflect a statistically significant improvement. Here, a maximal specificity of 100% is associated with a slight decrease in sensitivity (~35%). Conversely, if we hold sensitivity at 100%, the specificity levels are about 45%. Beyond 48 h, however, the biomarker’s AUROC further declines, as indicated by non-significant *p*-values (*p* > 0.05) (data not shown). This trend suggests that the diagnostic accuracy of pTau-217 diminishes in later post-injury phases, potentially due to the reduction of its levels or the influence of other physiological processes unrelated to acute injury.

### Glasgow outcome scale extended (GOSE) outcome analysis

We explored whether early CSF pTau-217 levels relate to 6-month neurological outcomes (as measured by GOSE). TBI patients were dichotomized into two groups: unfavorable (GOSE 1–4) and favorable (GOSE 5–8) outcomes. We compared pTau-217 levels in these groups at two early intervals: 6–24 h and 24–48 h post-injury (Supplementary Figure S9). The mean pTau-217 at 6–24 h was slightly higher in the favorable outcome group than in the unfavorable group, and a similar slight difference was seen at 24–48 h (favorable > unfavorable). However, these differences were not statistically significant (unpaired *t*-tests; 6–24 h: *p* = 0.418; 24–48 h: *p* = 0.271). The error bars (SEM) overlapped considerably between outcome groups, underscoring the lack of a clear separation. This somewhat counterintuitive trend (higher biomarker levels in better-outcome patients) suggests complexity in the relationship between acute tau release and long-term recovery. Given the small sample and exploratory nature of this analysis, these outcome observations are considered hypothesis-generating and not definitive. We interpret this finding with caution and propose that additional research with larger cohorts is needed to determine if early pTau-217 has any prognostic utility.

### Trajectory cluster analysis

**Figure 5:**
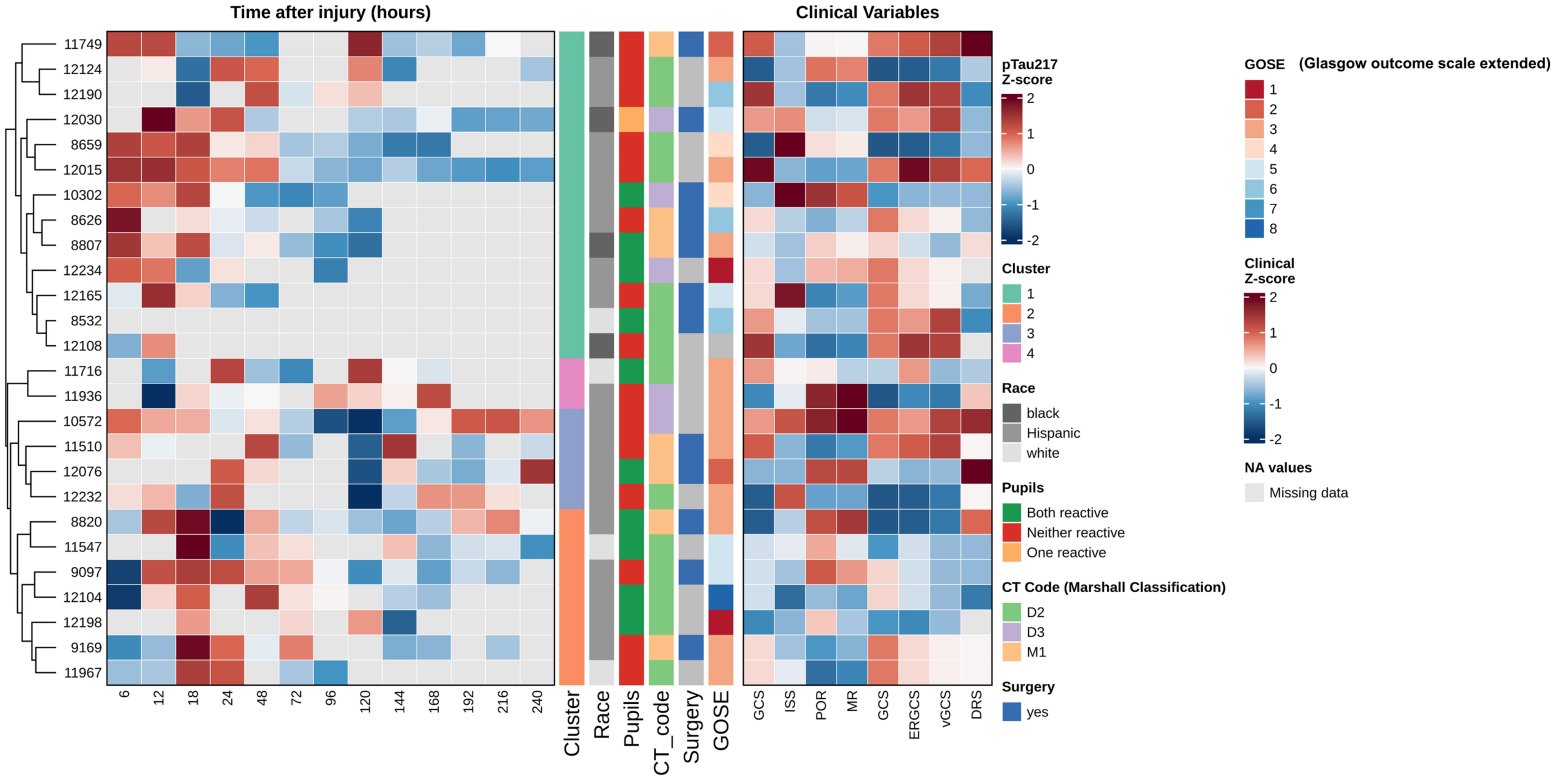
Hierarchical clustering of longitudinal CSF pTau-217 profiles. This figure presents a heatmap analysis of longitudinal CSF pTau-217 concentrations from 26 patients with severe TBI, providing biomarkers’ temporal dynamics and their relationship with clinical variables and outcomes. To facilitate comparison of dynamic patterns across patients, pTau-217 values at each time point (6–240 h post-injury) were *Z*-score normalized, with red indicating higher-than-average expression and blue indicating lower-than-average expression for a given patient. Gray cells represent missing data points. Patients were grouped using unsupervised hierarchical clustering (based on Euclidean distance and Ward’s linkage method), which organizes individuals based on the similarity of their entire longitudinal pTau-217 expression profile. This data-driven approach revealed four distinct patient clusters, each characterized by a unique temporal trajectory (e.g., sustained high elevation, early peak followed by rapid decline, or persistently low levels). An adjoining panel displays *Z*-score normalized clinical variables, allowing for a visual correlation between biomarker trajectories and clinical characteristics. Statistical analysis confirmed significant differences in mean pTau-217 levels between the identified patient clusters (ANOVA, *p* < 0.001), with Cluster 2, for example, exhibiting the highest sustained levels (mean *Z*-score = 1.42 ± 0.31). Crucially, this analysis reveals that the prognostic value of pTau-217 is embedded within its temporal pattern. A strong, statistically significant negative correlation was found between CSF pTau-217 levels at 24–48 h post-injury and the GOSE score at 6 months (Spearman’s *ρ* = −0.67, *p* < 0.01). This indicates that higher early CSF-pTau-217 levels are strongly associated with poorer long-term functional outcomes (lower GOSE scores). Furthermore, the biomarker profile was related to specific injury pathologies; patients with diffuse axonal injury, as defined by the Marshall Classification D3 (cisterns compressed or absent), showed significantly higher pTau-217 levels compared to those with focal lesions (M1) (two-tailed *t*-test, *p* = 0.023). This suggests that CSF-pTau-217 may be a specific marker of the widespread axonal damage characteristic of diffuse injuries. This sophisticated analytical approach, which focuses on individual longitudinal patterns, is more effective than analyses of group means at discrete time points (as shown in Supplementary Figure S9), highlighting the importance of personalized, trajectory-based biomarker interpretation in the heterogeneous TBI population.

To further investigate inter-patient variability in pTau-217 trajectories, we performed unsupervised hierarchical clustering of the 26 TBI patients based on their serial CSF pTau-217 profiles (6 h through 240 h). This analysis revealed four distinct clusters of patients with qualitatively different pTau-217 temporal patterns (Figure 5). Patients in Cluster 2 had consistently high and sustained pTau-217 levels across the acute phase; notably, this cluster’s average *Z*-score remained elevated at most time points. In contrast, patients in the other clusters (Clusters 1, 3, and 4) exhibited lower pTau-217 trajectories, with some displaying early peaks followed by rapid declines (e.g., Cluster 3), while others showed more moderate or fluctuating levels. Notably, these clusters appeared to correspond with specific clinical features: patients in Cluster 2 (high-pTau group) predominantly had diffuse axonal injury on their initial CT scans (Marshall classification grade D3) and tended to have worse 6-month outcomes. When we examined the CT findings and outcomes, patients with diffuse injury (D3) had significantly higher mean pTau-217 levels than those with focal contusions or mass lesions (Marshall grade M1) (two-tailed *t*-test, *p* = 0.023), aligning with the idea that more diffuse brain injury releases more tau. Additionally, CSF pTau-217 levels at 24–48 h showed an inverse correlation with GOSE score (Spearman *ρ* = −0.67, *p* < 0.01), indicating that patients with higher pTau-217 acutely often had poorer neurological outcomes at 6 months. These exploratory findings, visualized in Figure 5, suggest that a subset of severe TBI patients (Cluster 2) may experience an exceptionally robust and prolonged elevation of tau, potentially reflecting a greater axonal injury burden. In contrast, other patients exhibit a more transient or minimal release of tau. We stress that this clustering result is hypothesis-generating; nevertheless, it provides insight into the heterogeneity of TBI pathophysiology and raises the possibility that early pTau-217 trajectory patterns could be associated with injury type and prognosis.

## Discussion

Our findings demonstrate that CSF pTau-217 is acutely and transiently elevated following severe TBI. Within hours of injury, pTau-217 levels rise significantly (approximately three-fold above baseline), peaking in the first ~18–24 h, and then diminish over the ensuing days. This study echoes trends observed in Alzheimer’s disease (AD) biomarker research – for instance, the recent development of plasma pTau-217 assays for detecting AD pathology (19, 30, 50–52, 55–57). Rapid alterations in tau pathology are evident in acute TBI due to direct mechanical contact or trauma. The rapid reaction involves the excessive phosphorylation of tau proteins, which may lead to the formation of NFTs and contribute to sudden neuronal malfunction and death (58). In chronic neurodegenerative disorders such as AD, tau pathology advances at a slower pace, usually spanning many years. The process involves the gradual accumulation of hyperphosphorylated tau, leading to the formation of NFTs and extensive neuronal loss (59). Chronic neuroinflammation is often connected with gradual cognitive impairment, including memory loss and changes in behavior or personality. This comparison sheds light on the divergent and convergent pathways of tau pathology across acute and chronic brain injuries, offering insights into their mechanistic processes and potential therapeutic targets.

### Early detection and diagnostic utility

Total tau (T-tau) is elevated in plasma following TBI (60). Elevated T-tau levels have been associated with the severity of the injury and can be used as a biomarker to assess the extent of neuronal damage in acute settings. Our focus on pTau-217 extends this concept by examining a specific phosphorylated epitope of tau. Comparing pTau-217 with other tau antibodies, such as AT8 (pTau-181) and RZ3 (pTau-231), in cases of sTBI is crucial to understanding their diagnostic effectiveness and sensitivity in identifying early tau markers specific to TBI. The pTau-181 site was studied in TBI with varying results. Recent studies indicate that pTau-181 may not be significantly elevated in the plasma during the first year after msTBI, and its levels do not correlate with neuroimaging measures of neurodegeneration (61). This suggests that pTau-181 may not contribute to the progressive neurodegeneration commonly seen after TBI, at least within the first year after the injury.

Furthermore, pTau-181 dynamics in TBI are distinct from those observed in AD, where pTau-181 is a well-established marker (23). The lack of elevation over time and the absence of association with neurodegeneration markers in TBI patients highlight the limited utility of pTau-181 alone as a biomarker for ongoing neurodegenerative processes in TBI within this timeframe. pTau-202/205 has been reported as a prominent marker in the acute phase of TBI. The sensitivity of assays for pTau-202/205 in TBI is high, mainly when using the AT8 antibody, which is specific to these phosphorylation sites (62). The AT8 antibody is specific to pTau-202 and pTau-205. This specificity results from the antibody’s affinity for the epitope that includes both of these phosphorylation sites, enabling it to detect even small quantities of the modified protein. AT8 has been shown to detect picogram to nanogram levels of pTau. While AT8 shows high sensitivity, its specificity is less than ideal for TBI due to cross-reactivity with tau proteins involved in other neurodegenerative diseases such as AD.

pTau-231 shows more promise as a biomarker in TBI (63). Studies have demonstrated that pTau-231 levels can be elevated in the early stages of all severities following TBI, suggesting its role in the acute phase of the injury (19, 64, 65). pTau-231 has been identified as the most sensitive and specific CSF biomarker in distinguishing between chronic traumatic encephalopathy (CTE) and controls, as well as CTE and AD, suggesting its relevance in both acute and chronic phases of TBI (63). pTau-231 might have a prolonged elevation post-TBI, providing a wider diagnostic window than pTau-217. However, the early peak of pTau-217 observed in this study suggests that it might be superior for immediate post-injury assessments, offering critical insights before pTau-231 levels become detectable. TBI was reported to trigger a rapid acceleration of tau hyperphosphorylation, accelerating tauopathy, and long-term cognitive impairment (15). *Cis-*pTau-231 has been identified as a central mediator in TBI and neurodegeneration (66). Studies have shown that *cis*-pTau-231 is elevated in the early stages following TBI, suggesting its role in initiating the molecular cascade that leads to neurodegeneration (67–69). In human severe TBI (sTBI) and a rodent model of repetitive mild TBI during acute and subacute periods, serum pTau-231 was increased (63, 70, 71). Additionally, abnormal plasma pTau-231 elevation was reported in the chronic phase (6–18 months) after msTBI and in military members with repetitive TBI (72, 73).

pTau-396/404 (PHF-1 antibody) sites are also critical for tau pathology. pTau-396/404 promotes the detachment of tau protein from microtubules and its subsequent aggregation into paired helical filaments, the primary components of NFTs. In TBI, the acute mechanical impact and the resulting biochemical cascades can accelerate this phosphorylation, potentially leading to faster accumulation and spread of tau pathology. PHF-1’s utility as an early diagnostic marker is limited due to the delayed phosphorylation at these sites. Phosphorylation at Ser396/Ser404 may not peak until well after the initial injury (67, 74–77), making them less useful for early diagnostics but potentially valuable for assessing long-term neurodegeneration risks and progression toward CTE.

The rapid increase and subsequent decline in pTau-217 levels post-sTBI provide a sensitive and timely marker for initial injury assessment. The early peak of pTau-217, particularly when compared to the more gradual changes observed at other sites, highlights its potential to guide immediate therapeutic decisions. Moreover, the variability observed across different phosphorylation sites in response to TBI underscores the need for a panel of tau biomarkers, incorporating sites such as pTau-217 and pTau-231 for acute diagnostics and Ser396/Ser404 for long-term prognostics. Combining multiple phosphorylation sites could improve diagnostic accuracy and provide a more comprehensive view of TBI progression, from acute to chronic stages. This multiplex approach can help distinguish between different types of brain injuries and monitor therapeutic interventions.

Biomarkers of TBI are frequently assessed in body fluids, with most data derived from CSF or blood tests (78). We investigated CSF pTau as a biomarker in the early diagnostic stage of sTBI. This pattern illustrates the biomarker’s sensitivity to acute neuronal damage and the subsequent neuroinflammatory response triggered by TBI (79, 80). Such findings are reminiscent of the trajectory observed in AD biomarkers, where early pathological changes are detectable before clinical symptoms manifest, emphasizing the potential of pTau-217 in facilitating timely interventions (30, 56). Phosphorylation on multiple sites is considered a primary early event in the formation of tau aggregation. Thus, treatment can be started earlier, and severe symptoms may be avoided.

The exact mechanism and reasons underlying the hyperphosphorylation of tau upon TBI are yet to be entirely determined, but some potential explanations are available. TBI’s immediate impact and subsequent neuroinflammatory cascade may accelerate tau phosphorylation (Supplementary Figure S2), in contrast to AD’s slower, more progressive development of tau pathology. Axonal injury may initially cause tau-tau-microtubule dynamics to be perturbed by enabling its dissociation from microtubules, which could facilitate tau phosphorylation and aggregation into oligomers and NFTs following TBI (81). The chronic, persistent inflammatory response may exacerbate alterations of tau dynamics (82). Significantly, it has been found that immune activation contributes to the speeding progression of tauopathies through inflammatory cytokines such as interleukin-6 (IL-6) (83). Other cytokines, IL-1 and IL-1β, stimulate kinases that induce tau hyperphosphorylation (84–86). In addition, the misregulation of tau PTMs leads to tau aggregation directly or indirectly (87).

TBI, AD, and chronic traumatic encephalopathy (CTE) are under the umbrella of tauopathy (26, 88–90). TBI is linked with an increased risk of CTE^53^. There are similarities between AD and CTE in widespread neuronal loss associated with the deposition of tau. Tauopathies are diseases characterized by the same pathological hallmark: abnormal tau aggregation in brain cells. Compression of the cerebral cortex is standard in TBI (91). The findings of Asken et al. highlight the nuanced role of plasma pTau-181 and pTau-217 in distinguishing AD pathology within traumatic encephalopathy syndrome (TES) patients (29). Elevated pTau levels were predominantly associated with Aβ-positive TES cases, demonstrating specificity for AD-related tauopathies rather than CTE tau pathology. While plasma pTau-181 and pTau-217 provide insights into AD co-pathologies in TES, they failed to correlate with repetitive head trauma exposure or CTE-specific tau pathology. These observations align with our findings, emphasizing the need to contextualize pTau-217 dynamics within the unique pathophysiology of sTBI, rather than extrapolating from AD frameworks. Future research should strive to incorporate biomarker panels that consider the molecular diversity and temporal complexity of brain injuries, thus enhancing diagnostic accuracy and treatment approaches by utilizing pTau-217’s acute-phase sensitivity, especially in cases of sTBI. While the diagnosis of severe TBI itself typically relies on clinical assessments and imaging, the primary utility of early elevated CSF pTau-217 levels likely lies in their prognostic significance. Early changes in pTau-217 may reflect underlying neuropathological processes indicative of secondary neuronal injury, acute tau-mediated damage, and risk of long-term neurodegenerative sequelae. Thus, pTau-217 could potentially guide early stratification of patients according to risk, inform targeted therapeutic decisions, and aid in predicting long-term cognitive and neurological outcomes.

These findings suggest that early pTau-217 levels, measured within the first 48 h post-TBI, may not reliably predict long-term neurological outcomes as measured by GOSE scores. The lack of statistical significance at both evaluated intervals underscores the complexity of directly linking acute biomarker changes to clinical recovery trajectories. Although our cohort was part of the broader EPO Severe TBI trial, the present analysis was limited to a six-month outcome horizon. It did not specifically investigate longer-term neurodegeneration risks associated with acute pTau-217 elevations. Therefore, it remains unclear whether acute elevations in CSF pTau-217 have implications for the development of chronic neurodegenerative sequelae. Additionally, the limited sample size might have constrained statistical power, potentially obscuring subtle but clinically meaningful associations. Future studies with larger cohorts, extended longitudinal follow-up beyond 6 months, and detailed neuropathological assessments are essential to validate these preliminary observations and fully elucidate the prognostic value of pTau-217 in predicting TBI outcomes.

Although our data show an early surge in CSF pTau-217 among sTBI patients, these findings should be considered preliminary rather than definitive evidence of its singular value in diagnosing or monitoring TBI. Unlike the well-established role of pTau-217 in AD, where levels track a protracted neurodegenerative course, TBI elicits an acute and heterogeneous pathological cascade that may cause distinct tau phosphorylation and release patterns. Our observations suggest a potential window, within the first 48 h post-injury, when pTau-217 may aid in identifying acute neuronal stress; however, further validation is needed to establish its true clinical significance. More extensive longitudinal studies, spanning the immediate period of injury through long-term recovery, will be essential to clarify whether early elevations predict meaningful outcomes or therapeutic responses. Additionally, given the complexity of TBI, this biomarker is likely to have its greatest utility as part of a multiplex panel rather than as a standalone measure. Harnessing insights from tau-focused research in AD could catalyze new therapeutic avenues for TBI. However, doing so requires a careful, cross-disciplinary strategy that integrates molecular findings with clinical, imaging, and functional data. Ultimately, thorough validation and a holistic approach remain paramount before pTau-217 can be broadly applied in TBI care.

### Limitations and future directions

This study of pTau-217 as a biomarker for TBI has several limitations. First, our findings are derived from a relatively small, single-center cohort, which may limit generalizability. We did not have an independent validation cohort, so the results will require confirmation in larger studies. Future studies should include larger, multicenter cohorts to validate these findings and investigate how factors such as age, sex, and genetic background affect pTau-217 levels after TBI. Second, the control and TBI groups in our study were not perfectly demographically matched (for example, the TBI patients were, on average, younger). We included age as a covariate in our analyses and observed no significant impact of age on pTau-217 levels. Nevertheless, this difference between cohorts is a limitation and could introduce bias. A substantial limitation of this study is the difference in CSF collection methods between our cohorts; sTBI patient samples were collected from external ventricular drains, whereas control samples were obtained via lumbar puncture. It is well-established that a rostro-caudal concentration gradient exists for many proteins in the CSF, including tau. Specifically, studies have consistently shown that total tau and phosphorylated tau concentrations are higher in ventricular CSF compared to lumbar CSF. This suggests that the baseline pTau-217 levels in our lumbar-derived control samples may be systematically lower than those derived from a ventricular source (92–94). Consequently, the magnitude of the difference observed between our sTBI and control groups may be an underestimation of the actual effect. While this methodological difference is an explicit limitation, the fact that we still observed a highly significant elevation in pTau-217 in the sTBI cohort makes our findings more robust, as the comparison was inherently conservative. Future studies should ideally use source-matched CSF samples to provide a more precise quantification of this effect.

Temporal constraints pose another limitation: our study (like most TBI studies) focused on the acute phase post-injury. We have little information on how pTau-217 behaves in the subacute or chronic phases of TBI. The biomarker’s relevance beyond the first 10 days and its stability or secondary surges over time remain unknown. Additionally, individual biological variability is high; factors such as genetics (e.g., APOE genotype), co-morbid conditions, or extracranial injuries could affect tau levels. For example, we did not assess APOE status, which can influence blood–brain barrier integrity and TBI outcomes. We relied on donor screening to exclude significant neurological comorbidities in controls; however, unmeasured factors remain. This variability means that while pTau-217 shows promise, it should likely be used in combination with other markers (perhaps forming a panel that includes inflammatory or other injury markers) rather than alone. Moreover, our study did not include a detailed analysis correlating CSF biomarkers with neuroimaging findings. We observed, in a preliminary manner, that patients with diffuse axonal injury on CT tended to have higher acute pTau-217 levels; however, we did not systematically correlate pTau levels with MRI lesion load, diffusion imaging, or other advanced neuroimaging markers. This is a critical gap – future studies should examine if acute CSF pTau-217 correlates with imaging indicators of axonal injury or hemorrhage burden. Furthermore, ethical and practical considerations around CSF sampling must be acknowledged. Our study utilized CSF from EVDs in critically ill patients – a scenario where CSF access was part of clinical care. Lumbar puncture in less severe patients or controls is invasive and not always feasible. This motivates the exploration of less invasive biomarkers.

Encouragingly, recent advances show that pTau-217 can be measured in plasma with high sensitivity and correlates strongly with CSF levels in neurodegenerative disease contexts, accurately distinguishing amyloid-positive vs. amyloid-negative individuals (24, 29, 51, 55, 95). Although we did not measure plasma pTau-217 in the current study, this development holds promise for TBI: a blood-based pTau-217 assay could potentially obviate the need for routine CSF sampling, thereby greatly enhancing clinical applicability. We therefore suggest that future studies concurrently measure CSF and plasma pTau-217 in TBI patients to determine if blood levels mirror CSF changes and to assess whether plasma pTau-217 has diagnostic or prognostic utility in acute TBI. If plasma and CSF tau levels correlate well in TBI (as they do in AD), a plasma pTau-217 test could serve as a safer, rapid screening tool in the emergency or critical care setting. Another limitation relates to our assay itself: we used a prototype direct ELISA. While this allowed us to detect differences between groups, its quantitative precision was limited. Our prototype ELISA’s standard curve had an R^2^ of 0.843, indicating a suboptimal fit; accordingly, the absolute concentrations reported should be interpreted with caution as approximate values. We have since pursued the development of a more sensitive sandwich ELISA for pTau-217. Indeed, the application of an improved pTau-217 assay format (e.g., high-sensitivity sandwich ELISA or SIMOA) that is compatible with both CSF and blood will be critical for the next phase of research.

A more sensitive assay could detect lower pTau-217 levels and improve linear quantitation, thereby enhancing the biomarker’s reliability. Looking ahead, longitudinal studies extending beyond the acute phase are needed to understand how long pTau-217 remains elevated and whether secondary rises occur (for example, in the event of complications like swelling or secondary insults). It will also be essential to determine if acute pTau-217 elevation has any predictive value for long-term outcomes such as cognitive function or risk of chronic neurodegeneration (e.g., post-traumatic encephalopathy). While our outcome analysis did not show a clear relationship, it was underpowered; we consider those analyses hypothesis-generating. Future research with larger cohorts should explicitly test whether early CSF pTau-217 (or its trajectory, as suggested by our cluster analysis) correlates with neurological outcomes at 6–12 months or beyond. Such studies should also incorporate comprehensive neuroimaging and perhaps fluid biomarkers of other pathways (like neuroinflammation or neurodegeneration) to build a more complete picture. It may be that multivariate models including pTau-217, other biomarkers (e.g., NF-L, GFAP, UCH-L1), and clinical variables will be required to robustly predict outcomes in TBI. Finally, integrating a biomarker like pTau-217 into clinical practice will require standardization and validation. As we discussed, blood-based assays could be a game-changer in terms of practicality. Standard operating procedures for sample handling, assay calibration, and interpretation thresholds would need to be established. Our study, while preliminary, provides a foundation that early CSF pTau-217 rises are a real phenomenon in severe TBI. The following steps will clarify whether this finding can be translated into a useful clinical tool, for instance, to identify patients with diffuse axonal injury or to monitor their response to neuroprotective therapies (should any emerge).

## Conclusion

In conclusion, our study demonstrates for the first time that CSF pTau-217 is significantly elevated during the acute phase following severe TBI, peaking within the first 18–24 h and returning toward baseline thereafter. CSF pTau-217 showed robust diagnostic separation of sTBI patients from healthy controls in the first 48 h post-injury, suggesting that pTau-217 may be a promising biomarker for acute neuronal injury. The notable inter-patient variability, highlighted by our trajectory clustering analysis, underscores the complexity and heterogeneity of tau responses to brain trauma. However, these findings must be interpreted cautiously given the modest sample size and exploratory nature of our analysis. Future studies with larger cohorts, extended longitudinal sampling, and improved assays (including blood-based measurements) are essential to validate pTau-217 as a reliable biomarker for TBI. Integrating pTau-217 with clinical assessments, neuroimaging, and other biomarkers may ultimately refine diagnostic and prognostic models for TBI. Ultimately, it remains to be determined whether early CSF pTau-217 elevations can predict long-term outcomes or neurodegenerative sequelae – at present, our outcome correlations are preliminary. Establishing such a link would open the door to pTau-217 being used not only for injury detection but also for prognostication and possibly as a pharmacodynamic marker in therapeutic trials. We view our findings as an essential first step in that direction, with much work ahead to bring pTau-217 into clinical application for TBI management.

### Transparency, rigor, and reproducibility summary

This study, which focuses on the temporal dynamics of pTau-217 in patients with severe TBI, adhered to rigorous standards for transparency and reproducibility. Pre-registration was not applicable because of the retrospective analysis. The analytic plan, developed before data analysis, involved a comprehensive statistical approach utilizing GraphPad Prism for variance analysis and ROC curve assessment, ensuring the high reliability of the findings. Our sample size was determined by the availability of archived samples, with detailed demographic characteristics provided for TBI and control subjects. Blinding was maintained during ELISA and WB analyses to minimize bias. Biofluid sample handling and analysis protocols were strictly followed, ensuring consistency across all samples. The primary fluid biomarker analyses were validated through repeated measures and control comparisons, emphasizing the specificity of pTau-217 detection. Statistical tests were selected based on the data distribution, taking into account the handling of outliers and missing data. Our study supports pTau-217 as a significant biomarker for early diagnosis of TBI, providing a foundation for future research and clinical applications. Data sharing arrangements comply with institutional IRB standards, with de-identified data available upon request to ensure further validation and transparency.

## Data Availability

The original contributions presented in the study are included in the article/Supplementary material, further inquiries can be directed to the corresponding authors.

## Supplementary material

SUPPLEMENTARY FIGURE S1 Experimental workflow of the direct ELISA for pTau-217 detection. This flowchart outlines the key steps, reagents, and incubation times for the direct ELISA protocol used for quantifying pTau-217 in human CSF samples from severe traumatic brain injury (sTBI) patients. The figure illustrates the steps and conditions for the assay based on the enzyme-linked immunosorbent assay (ELISA) principle. ELISA is a technique that uses antibodies and color change to identify and quantify substances in a liquid sample. The figure also indicates the number and type of samples used in the assay, as well as the wavelengths at which the optical density (OD) values were measured. The OD values are used to determine the presence and the level of pTau-217 in the samples. pTau-217 is a phosphorylated form of tau protein that is a potential biomarker for TBI and Alzheimer’s disease. The key steps of the assay are numbered sequentially. (1) The process begins by coating the ELISA plate wells with CSF samples diluted to a total protein concentration of 10 μg/mL and incubating overnight (O/N, 16 h) at 4°C to allow for the passive adsorption of proteins, including pTau-217, to the solid phase. (2) After washing to remove unbound material, a primary antibody specific for pTau-217, conjugated to an enzyme (horseradish peroxidase), is added at a concentration of 1 μg/mL and incubated overnight at 4°C. (3) Following a series of washes and addition of secondary antibody, (4) a chromogenic substrate (1 mg/mL) is added. The enzyme conjugated to the antibody catalyzes a reaction that converts the substrate into a colored product. (5) The initial color development is measured as optical density (OD) at 600 nm. (6) The reaction is then terminated by the addition of a stop solution (2 M sulfuric acid), which stabilizes the color. (7) The final, stable absorbance is read at 450 nm. The intensity of the color, quantified by the OD reading, is directly proportional to the amount of pTau-217 present in the original CSF sample.

SUPPLEMENTARY FIGURE S2 Schematic of tau protein phosphorylation sites implicated in TBI. This figure provides a schematic representation of the longest human brain isoform of the tau protein, 2N4R (441 amino acids), to contextualize the pTau-217 biomarker within the broader landscape of tau post-translational modifications associated with traumatic brain injury (TBI). Tau is a microtubule-associated protein crucial for neuronal stability and axonal transport. In several neurodegenerative disorders, including TBI-related neurodegeneration, tau becomes abnormally hyperphosphorylated, causing it to detach from microtubules and aggregate into neurofibrillary tangles (NFTs), a key pathological hallmark of these conditions. The diagram illustrates the primary functional domains of the 2N4R tau isoform. The N-terminus contains the projection domain with two inserts (N1 and N2). This is followed by a proline-rich domain, which is heavily involved in signaling interactions. The core of the protein consists of the microtubule-binding domain, which in this isoform contains four repeat regions (R1, R2, R3, and R4) that mediate its primary function of stabilizing microtubules. A C-terminal flanking region completes the structure. Highlighted in blue are numerous amino acid residues that have been reported in the scientific literature to undergo phosphorylation following TBI. These include sites such as Tyrosine-18 (pTyr18), Serine-199 (pS199), Serine-202/Threonine-205 (targeted by antibodies CP13 and AT8), Threonine-181 (AT270), Threonine-231 (RZ3), and Serine-396/Serine-404 (PHF-1), among others. The specific antibodies used to detect these phosphorylated epitopes are indicated in red. The phosphorylation site of primary interest in the present study, Threonine-217 (pTau-217), is prominently demarcated by a dashed red box. While pTau-217 has emerged as a particular and sensitive biomarker for Alzheimer's disease (AD) pathology, correlating strongly with amyloid and tau PET imaging 16, its role in the acute phase of TBI is less understood. The elevation of pTau-217 following TBI raises a fundamental question central to this investigation: does this biomarker reflect a transient, acute injury response, or does it signify the initiation of a chronic, AD-like neurodegenerative cascade. This schematic illustrates that pTau-217 is one of many pathological modifications to tau following TBI, positioning this study as an inquiry into the specific mechanisms linking acute traumatic injury to the potential for long-term neurodegeneration.

SUPPLEMENTARY FIGURE S3 Titration and optimization of anti-pTau-217 antibody for direct ELISA. This figure presents the results of the systematic optimization of the anti-pTau-217 antibody concentration, a crucial step in ensuring the sensitivity, specificity, and reliability of the direct ELISA method. The optimization was performed using three distinct substrates to validate the antibody's performance against both purified and biologically complex forms of tau protein. Data points represent the mean ± standard error of the mean (SEM) from three independent experiments (*n* = 3), and statistical significance was assessed using a two-way ANOVA, which confirmed a highly significant effect of antibody concentration (*p* < 0.001). **(A)** Dose-response curve: this panel illustrates the relationship between increasing antibody dilutions (from 1:50000 to 1:100, corresponding to concentrations from 0.02 to 10.00 μg/mL) and the resulting absorbance signal (OD450). The assay was tested against three immobilized substrates: recombinant unphosphorylated Tau-441 protein (20 ng), serving as a negative control; dual-kinase (GSK3β+DYRK1A)-phosphorylated Tau-441 (20 ng), serving as a precise positive control for pTau-217; and lysates from SH-SY5Y neuroblastoma cells treated with the phosphatase inhibitor okadaic acid (OA, 100 nM), representing a complex biological sample with induced hyperphosphorylation. As expected, the signal increases with antibody concentration for all substrates, with the strongest response observed for the kinase-phosphorylated tau. **(B)** Assay sensitivity: this panel displays the calculated pTau-217 concentrations (ng/mL) derived from standard curves generated at each antibody dilution. The results demonstrate that higher antibody concentrations enable the detection and quantification of higher levels of pTau-217, indicating a direct relationship between antibody concentration and the effective dynamic range and sensitivity of the assay. **(C)** Signal-to-noise ratio: this panel presents the most critical parameter for determining the optimal antibody concentration. The signal-to-noise (S/N) ratio was calculated by dividing the signal from the positive control samples (kinase-phosphorylated tau and OA-treated cell lysate) by the signal from the negative control (unphosphorylated Tau-441). A higher S/N ratio indicates greater assay specificity and a better ability to distinguish a valid signal from background noise. The analysis shows that the S/N ratio improves significantly with increasing antibody concentration, reaching a plateau or peak efficiency at the 1:100 dilution (10.00 μg/mL). While the absolute signal continued to grow in panels **(A,B)**, this analysis reveals that beyond this point, further increases in antibody concentration add proportionally more background noise, thus reducing assay efficiency. Therefore, the 1:100 dilution was selected as the optimal concentration for all subsequent experiments, providing the best balance of sensitivity and specificity.

SUPPLEMENTARY FIGURE S4 Association of age with CSF pTau-217 levels across post-injury time points. This figure systematically investigates the potential confounding influence of patient age on CSF pTau-217 levels measured longitudinally after severe TBI. The analysis is presented as a series of 13 subplots, each corresponding to a specific time point from 6 to 240 h post-injury. Each subplot displays individual patient data as a scatter plot of age versus pTau-217 level, with a linear regression line fitted to visualize the trend. To assess the strength and significance of any monotonic relationship between age and pTau-217, Spearman correlation analysis was performed for each time point. The Spearman correlation coefficient (ρ) and the corresponding *p*-value are displayed above each subplot. The key finding from this comprehensive analysis is the consistent absence of a statistically significant correlation between age and pTau-217 levels at every measured time point (all *p* > 0.05). The Spearman ρ values were consistently low, ranging from −0.139 to 0.004, indicating no meaningful association. This is a crucial adverse finding that has important implications for the interpretation of pTau-217 as a TBI biomarker. In the context of neurodegenerative diseases, particularly Alzheimer's disease (AD), plasma and CSF pTau-217 levels are known to be strongly associated with amyloid pathology, which itself is highly age-dependent. The lack of an age correlation in this acute TBI cohort suggests that the mechanisms driving the release of pTau-217 in the immediate aftermath of injury are distinct from those underlying chronic, age-related neurodegeneration. This finding supports the hypothesis that the observed elevation of pTau-217 is a direct result of the acute traumatic pathophysiology, rather than a reflection of pre-existing, age-dependent pathology. This demonstrates due diligence in assessing a major potential confounder and strengthens the case for pTau-217 as a specific biomarker of the acute injury process itself.

SUPPLEMENTARY FIGURE S5 Distribution of CSF pTau-217 levels across time points by race and ethnicity. This figure illustrates the longitudinal dynamics of CSF pTau-217 concentrations stratified by patient-identified race and ethnicity (White, Black, and Hispanic). The visualization employs a combination of box plots and individual data points to provide a comprehensive view of both group-level trends and within-group variability over the first 240 h post-injury. Boxplots depict the median, interquartile range (IQR), and whiskers (extending to 1.5 times the IQR) for each group at each time point. Overlaid circular dots represent individual patient measurements, allowing for an assessment of data distribution and potential outliers. Statistical analysis was conducted using one-way Analysis of Variance (ANOVA) at each time point to compare the mean pTau-217 levels among the three racial/ethnic groups. The resulting *p*-values are displayed above the plots. A red asterisk (*) denotes time points where the ANOVA test yielded a statistically significant difference (*p* < 0.05). The analysis reveals that while pTau-217 levels were not significantly different among the groups during the early acute phase, statistically significant disparities emerged at later time points, specifically at the 168–192 h (*p* = 0.006), 192–216 h (*p* = 0.023), and 216–240 h (p = 0.031) intervals. This is a novel and potentially important observation, as the literature indicates that significant racial and ethnic disparities exist in TBI incidence and outcomes. However, the biological underpinnings remain poorly understood. However, this finding must be interpreted with caution. The sample sizes for the Black (*n* = 5) and Hispanic (*n* = 6) patient groups are small, which limits the statistical power and generalizability of the results. Therefore, this analysis should be considered exploratory and hypothesis-generating in nature. The observed differences could arise from multiple factors, including underlying biological or genetic variations that influence the neuroinflammatory or neurodegenerative response to injury, or unmeasured social determinants of health that can impact injury circumstances and clinical care pathways. This finding emphasizes a key area for future research, highlighting the importance of studying larger, more diverse, and well-characterized groups to confirm these results and understand the mechanisms behind possible racial and ethnic differences in biomarker responses after TBI.

SUPPLEMENTARY FIGURE S6 Correlation between admission GCS scores and CSF pTau-217 levels. This scatter plot investigates the relationship between the severity of impaired consciousness at admission, as measured by the GCS, and CSF pTau-217 concentrations measured 24–48 hours post-injury. The GCS is a fundamental clinical tool where lower scores indicate a more severe impairment of consciousness. Each point on the plot represents an individual patient. Patients who were intubated at the time of GCS assessment, precluding a verbal response, are marked with a red “T”. For these cases, the verbal component of the GCS was imputed based on clinical judgment, a standard practice in critical care. Linear regression analysis of these data yielded a correlation coefficient (*r*) of 0.436 with a *p*-value of 0.054. This result indicates a moderate positive trend that approaches, but does not reach, the conventional threshold for statistical significance. The direction of this trend is paradoxical; it suggests that patients with less severe impairment of consciousness (higher GCS scores) tended to have higher levels of CSF pTau-217. This counterintuitive finding suggests that pTau-217 is not a simple, linear marker of the global brain dysfunction captured by the GCS. Several mechanisms could explain this observation. First, pTau-217 may reflect a specific type of pathophysiology, such as diffuse axonal injury, which does not correlate perfectly with the global functions (eye, verbal, motor responses) assessed by the GCS. A patient may have significant axonal pathology while retaining some functions, resulting in a higher GCS score. Second, in patients with the most severe injuries (i.e., very low GCS scores), widespread tissue destruction and severe disruption of the blood-brain barrier could lead to altered biomarker clearance dynamics. Instead of accumulating in the CSF, the biomarker might be rapidly cleared into the peripheral circulation or degraded in situ, paradoxically resulting in lower measured CSF concentrations. This complex relationship highlights the unique pathophysiological information provided by pTau-217, which differs from traditional clinical severity scales. It underscores the need to move beyond a simplistic "more is worse" interpretation for this biomarker.

SUPPLEMENTARY FIGURE S7 Distribution of admission GCS scores in early vs. late CSF sampling cohorts. This figure shows a comparative histogram analysis of GCS scores for two patient subgroups: “Early Contributors,” who provided CSF samples within the first 24 h after injury (blue bars), and “Late Contributors,” who provided samples during the later 96–240 hour period (orange bars). This analysis aims to evaluate the stability of the study cohort over time and examine potential selection or survivor bias, a common issue in long-term studies of critically ill patients. The histograms reveal a significant change in the composition of the patient cohort over time. The Early Contributor group exhibits a broad distribution of GCS scores, including a substantial number of patients with very severe injuries (GCS ≤ 6). This reflects the initial heterogeneity of the sTBI population admitted to the intensive care unit. In stark contrast, the Late Contributor group is heavily skewed toward patients with higher admission GCS scores (predominantly GCS ≥ 8), indicating less severe initial injuries or more favorable recovery trajectories. Patients with the most severe initial injuries are largely absent from this later cohort. This observation provides strong evidence of survivor bias. Patients with the most devastating injuries (lowest GCS scores) are less likely to be available for sampling at later time points, often due to mortality or clinical instability that precludes procedures like lumbar puncture. This evolving cohort composition has profound implications for the interpretation of longitudinal biomarker data. A simple analysis of group-mean biomarker levels over time could be highly misleading; an observed decrease in the average pTau-217 level might not reflect an actual biological process of recovery within individuals, but rather an analytical artifact caused by the selective attrition of patients with the highest initial biomarker levels. This methodological insight highlights the limitations of basic longitudinal analyses. It offers a strong justification for using more advanced, individual-focused analytical methods, such as the hierarchical clustering of patient trajectories shown in Supplementary Figure S4, which are less vulnerable to this kind of bias.

SUPPLEMENTARY FIGURE S8 Correlation between injury severity score (ISS) and CSF pTau-217 levels at early time intervals. This figure presents a multi-panel analysis exploring the relationship between a patient’s overall burden of systemic trauma, quantified by the Injury Severity Score (ISS), and the concentration of CSF pTau-217 at four distinct time intervals in the early post-injury phase: **(A)** 0–6 h, **(B)** 6–12 h, **(C)** 12–18 h, and **(D)** 24–48 h. The ISS is an anatomical scoring system based on the Abbreviated Injury Scale (AIS) that assesses the severity of injuries across six body regions, providing a measure of the patient's total systemic trauma rather than just the brain injury. Each scatter plot displays individual patient data, along with a fitted linear regression line, to visualize the trend. The Pearson correlation coefficient (*r*) and its corresponding *p*-value are provided for each time interval to assess the strength and statistical significance of the linear association. Across all four time intervals, the observed correlations are weak (*r* values ranging from 0.25 to 0.39) and fail to reach statistical significance (all *p* > 0.05). For example, at 0–6 h, the correlation was *r* = 0.38 (*p* = 0.122), and at 24–48 h, it was *r* = 0.25 (*p* = 0.293). The consistent lack of a significant correlation between ISS and CSF pTau-217 is a critical finding that emphasizes the brain-specific nature of this biomarker. It provides strong evidence that the elevation of pTau-217 in the CSF is a direct consequence of neuropathological processes occurring within the central nervous system (CNS) and is not merely a reflection of the overall severity of systemic, extracranial injuries. This organ specificity is a desirable characteristic for a neurological biomarker, as it suggests that pTau-217 levels are not confounded by polytrauma. This finding enhances the potential utility of pTau-217 for specifically monitoring CNS injury and the response to neuroprotective, CNS-targeted therapies, distinguishing its signal from the systemic inflammatory response to trauma.

SUPPLEMENTARY FIGURE S9 Comparison of early (0–48 h) CSF pTau-217 levels with 6-month Glasgow outcome scale-extended (GOSE) scores. This figure assesses the prognostic value of early CSF pTau-217 levels by comparing average values in patients with positive versus negative long-term outcomes. Patients were divided into two groups based on their 6-month GOSE score: an “Unfavorable” group (GOSE 1–4, indicating death to severe disability) and a “Favorable” group (GOSE 5–8, indicating moderate disability to good recovery). The analysis focused on two early post-injury periods: **(A)** 6–24 h and **(B)** 24–48 h. Bar graphs display the average pTau-217 levels for each outcome group, with error bars indicating the standard error of the mean (SEM). Statistical differences were examined using unpaired t-tests to determine if the mean pTau-217 levels differed significantly between the outcome groups. Results reveal that in both time intervals, there was no significant difference in early pTau-217 levels between patients with favorable and unfavorable outcomes. For the 6–24 h window, the *p*-value was 0.418, and for the 24–48 h window, it was 0.271. Although this non-significant result seems to contradict the strong prognostic link for pTau-217 found in Figure 5, it highlights an important methodological issue in biomarker analysis. The absence of significance here is likely due to several factors that lower analytical power. First, splitting a continuous or ordinal scale, such as GOSE, into two groups loses detailed information and can hide more subtle relationships. Second, this analysis relies on static, single-time-point measurements, which overlook the dynamic information within the biomarker's complete temporal profile —a feature shown to be prognostically important in Figure 5. Third, the statistical test used, a *T*-test comparing group means, is less sensitive to complex associations than the correlation and clustering methods used elsewhere. Lastly, these data are from CSF, while the strongest prognostic signal was seen in plasma, which may have different release kinetics and clearance pathways. Therefore, this figure does not necessarily mean pTau-217 lacks prognostic value, but shows that simple analyses of early CSF measurements don't fully capture its usefulness. It highlights the benefit of a more detailed approach that considers the biomarker's complete longitudinal profile.
