# Supplementary material for: Early cerebrospinal fluid elevations of pTau-217 in severe traumatic brain injury subjects
Source: Front Neurol. 2025 Jul 30;16:1632679. doi: 10.3389/fneur.2025.1632679 (PMC12344560; doi:10.3389/fneur.2025.1632679)
Supplement: SUPPLEMENTARY FIGURE S1 — Experimental workflow of the direct ELISA for pTau-217 detection. This flowchart outlines the key steps, reagents, and incubation times for the direct ELISA protocol used for quantifying pTau-217 in human CSF samples from severe traumatic brain injury (sTBI) patients. The figure illustrates the steps and conditions for the assay based on the enzyme-linked immunosorbent assay (ELISA) principle. ELISA is a technique that uses antibodies and color change to identify and quantify substances in a liquid sample. The figure also indicates the number and type of samples used in the assay, as well as the wavelengths at which the optical density (OD) values were measured. The OD values are used to determine the presence and the level of pTau-217 in the samples. pTau-217 is a phosphorylated form of tau protein that is a potential biomarker for TBI and Alzheimer’s disease. The key steps of the assay are numbered sequentially. (1) The process begins by coating the ELISA plate wells with CSF samples diluted to a total protein concentration of 10 μg/mL and incubating overnight (O/N, 16 h) at 4°C to allow for the passive adsorption of proteins, including pTau-217, to the solid phase. (2) After washing to remove unbound material, a primary antibody specific for pTau-217, conjugated to an enzyme (horseradish peroxidase), is added at a concentration of 1 μg/mL and incubated overnight at 4°C. (3) Following a series of washes and addition of secondary antibody, (4) a chromogenic substrate (1 mg/mL) is added. The enzyme conjugated to the antibody catalyzes a reaction that converts the substrate into a colored product. (5) The initial color development is measured as optical density (OD) at 600 nm. (6) The reaction is then terminated by the addition of a stop solution (2 M sulfuric acid), which stabilizes the color. (7) The final, stable absorbance is read at 450 nm. The intensity of the color, quantified by the OD reading, is directly proportional to the amount of pTau-217 present in the [file Data_Sheet_1.pdf]

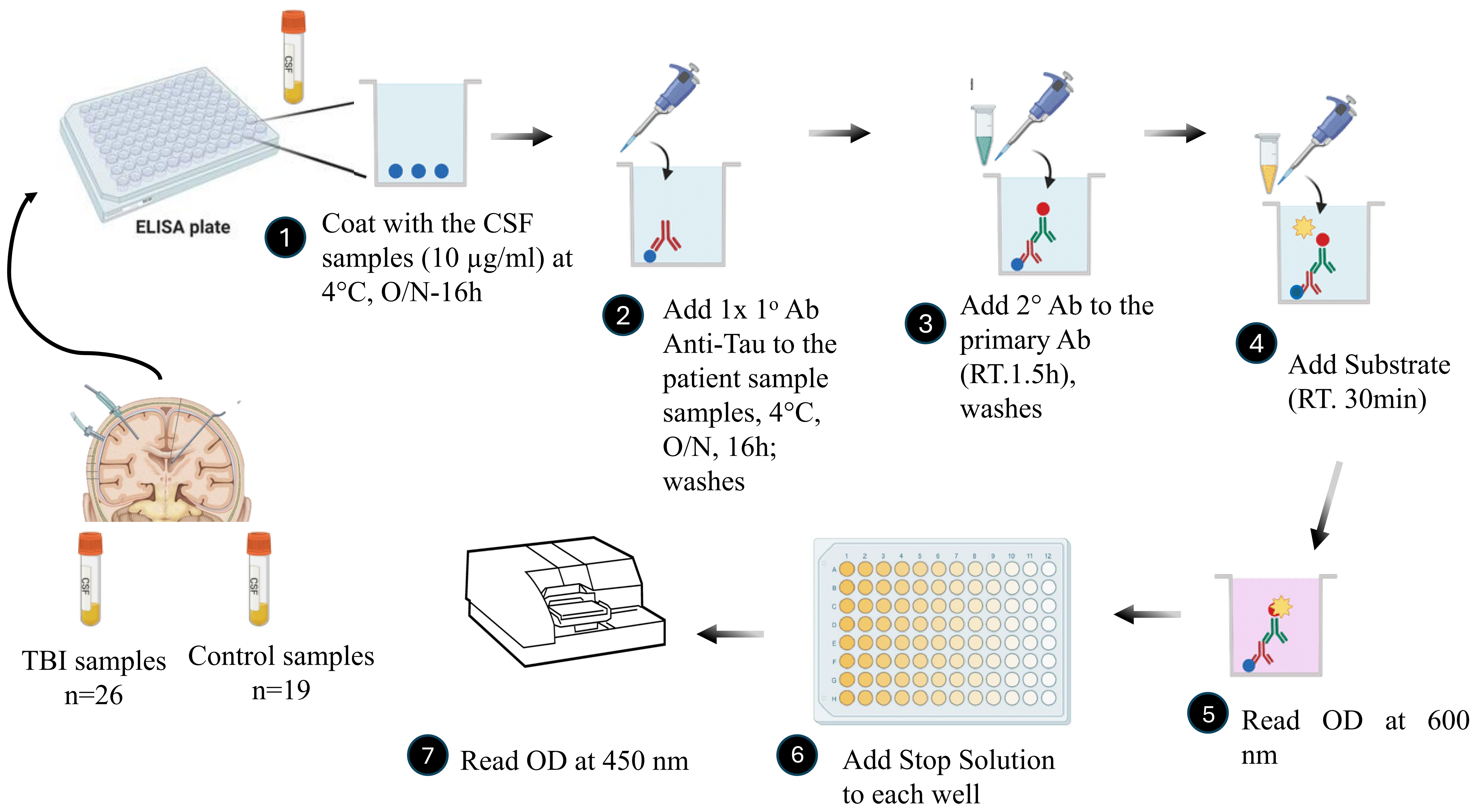

ELISA plate
CSF
1 Coat with the CSF samples (10 µg/ml) at 4°C, O/N-16h
2 Add 1x 1° Ab Anti-Tau to the patient sample samples, 4°C, O/N, 16h; washes
3 Add 2° Ab to the primary Ab (RT.1.5h), washes
4 Add Substrate (RT. 30min)
5 Read OD at 600 nm
6 Add Stop Solution to each well
7 Read OD at 450 nm
TBI samples n=26
Control samples n=19

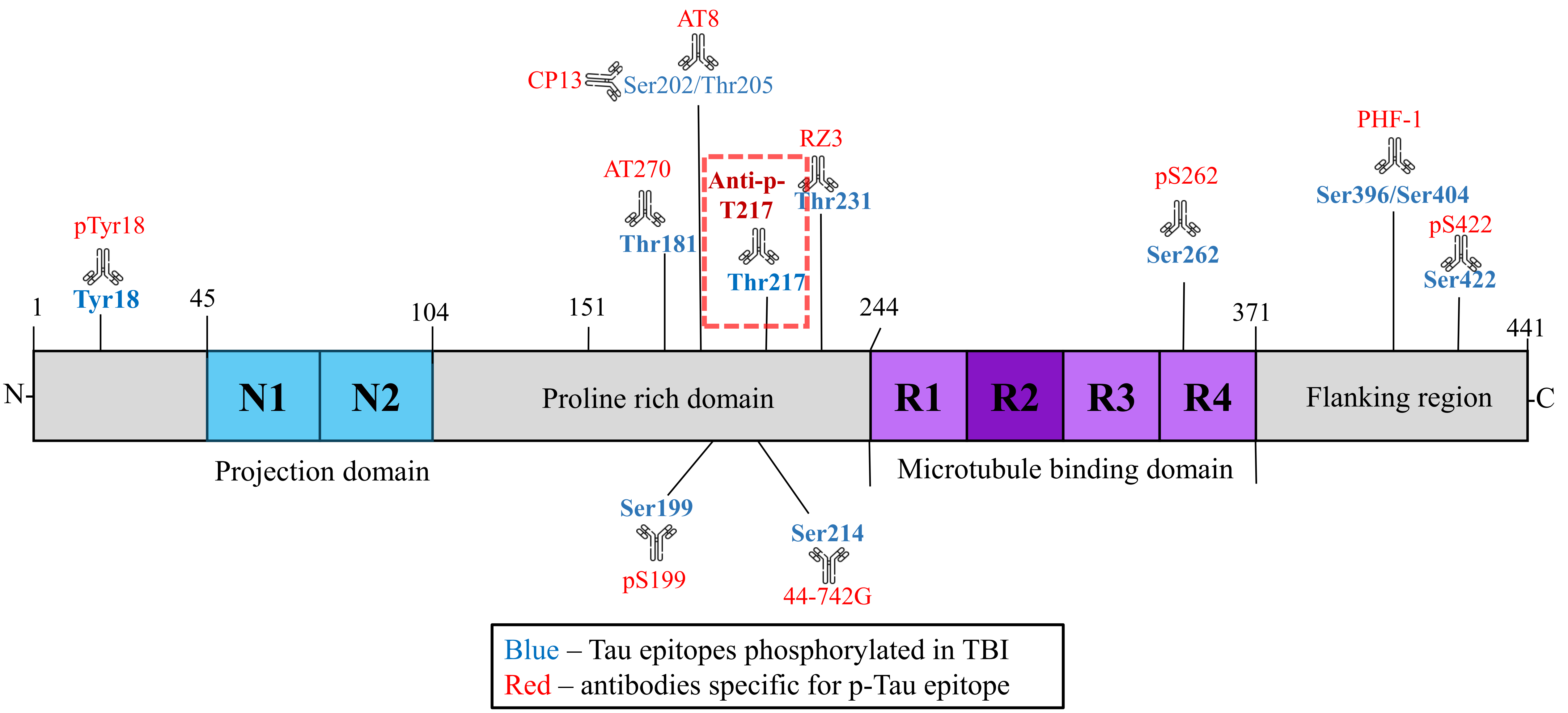
AT8
CP13
Ser202/Thr205
RZ3
PHF-1
AT270
Anti-p-T217
Thr231
pS262
Ser396/Ser404
pTyr18
Thr181
Thr217
Ser262
pS422
Ser422
Tyr18
1
45
104
151
244
371
441
N
N1
N2
Proline rich domain
R1
R2
R3
R4
Flanking region
C
Projection domain
Microtubule binding domain
Ser199
pS199
Ser214
44-742G
Blue – Tau epitopes phosphorylated in TBI
Red – antibodies specific for p-Tau epitope

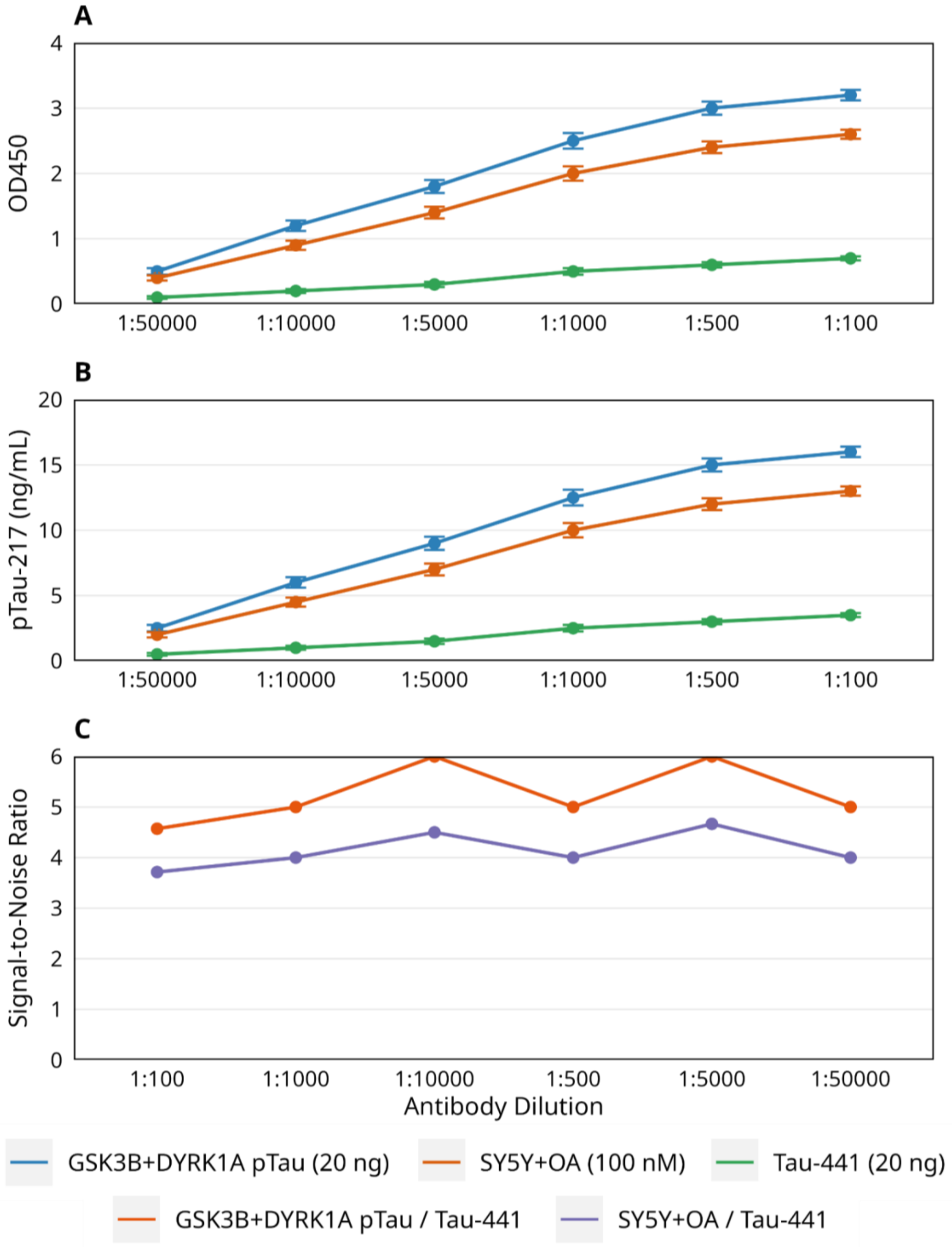

A
OD450
4
3
2
1
0
1:50000
1:10000
1:5000
1:1000
1:500
1:100
B
pTau-217 (ng/mL)
20
15
10
5
0
1:50000
1:10000
1:5000
1:1000
1:500
1:100
C
Signal-to-Noise Ratio
6
5
4
3
2
1
0
1:100
1:1000
1:10000
1:500
1:5000
1:50000
Antibody Dilution
GSK3B+DYRK1A pTau (20 ng)
SY5Y+OA (100 nM)
Tau-441 (20 ng)
GSK3B+DYRK1A pTau / Tau-441
SY5Y+OA / Tau-441

Age vs. P-tau Levels Across Time Points with Spearman Correlation

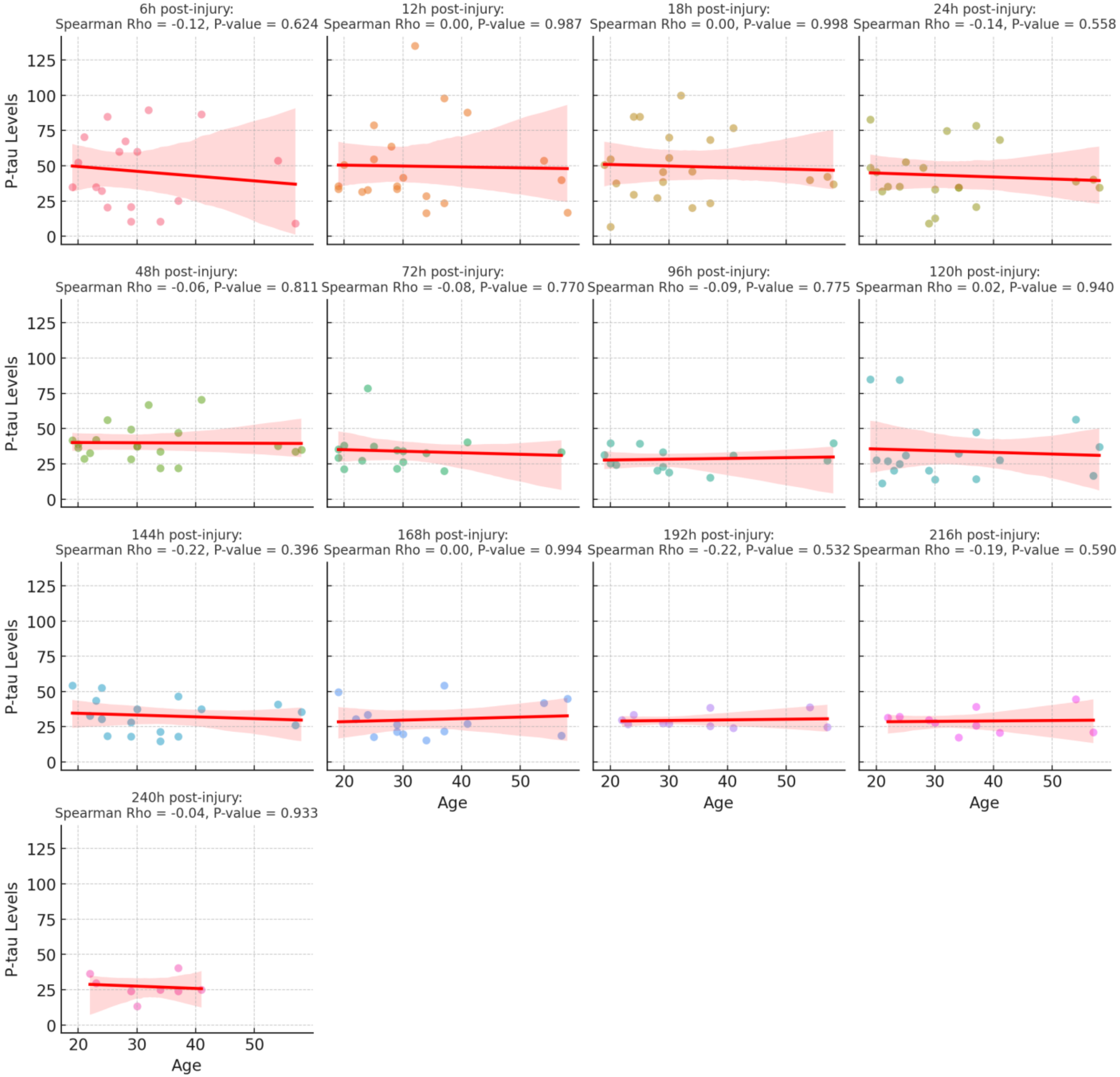

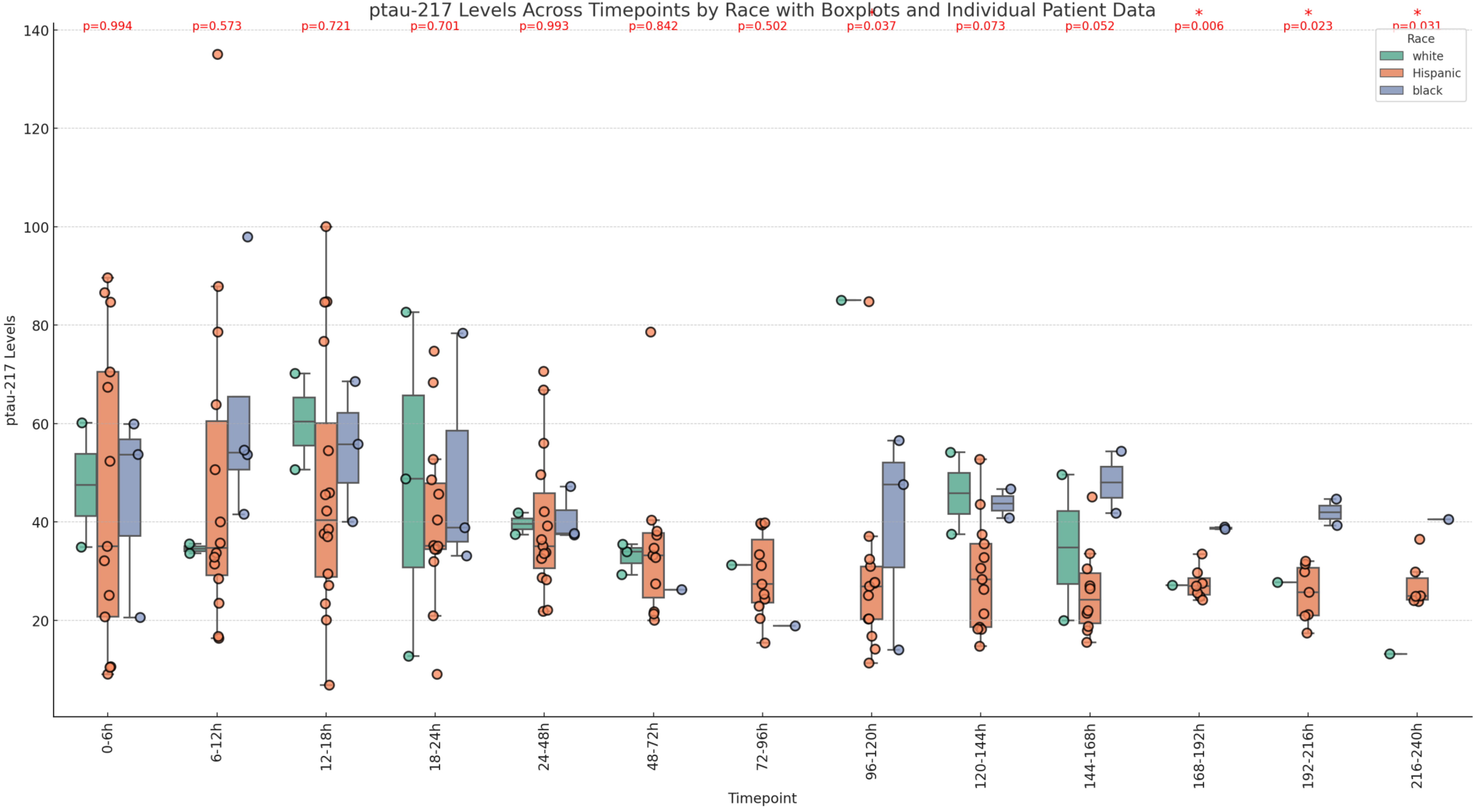

ptau-217 Levels Across Timepoints by Race with Boxplots and Individual Patient Data
p=0.994
p=0.573
p=0.721
p=0.701
p=0.993
p=0.842
p=0.502
p=0.037
p=0.073
p=0.052
p=0.006
p=0.023
p=0.031
Race
white
Hispanic
black
ptau-217 Levels
Timepoint
0-6h
6-12h
12-18h
18-24h
24-48h
48-72h
72-96h
96-120h
120-144h
144-168h
168-192h
192-216h
216-240h

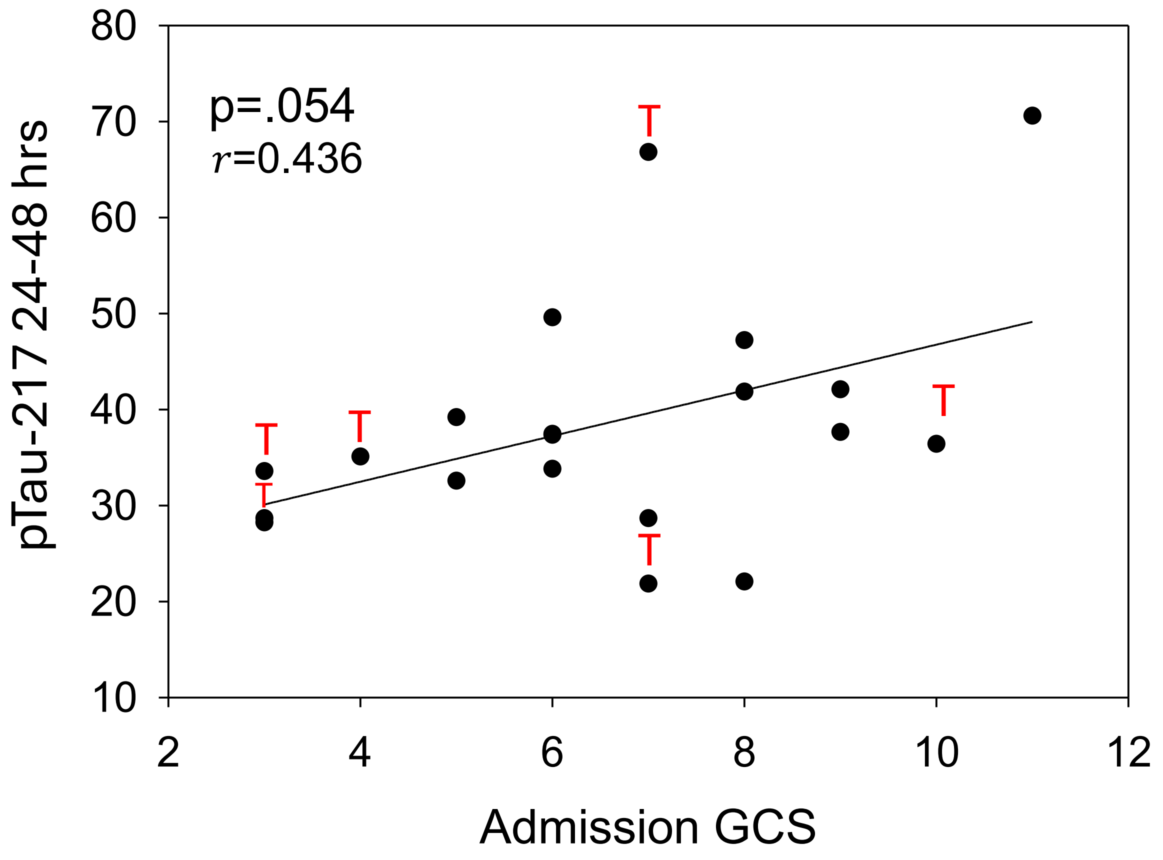

p=.054
r=0.436
pTau-217 24-48 hrs
Admission GCS
80
70
60
50
40
30
20
10
2
4
6
8
10
12

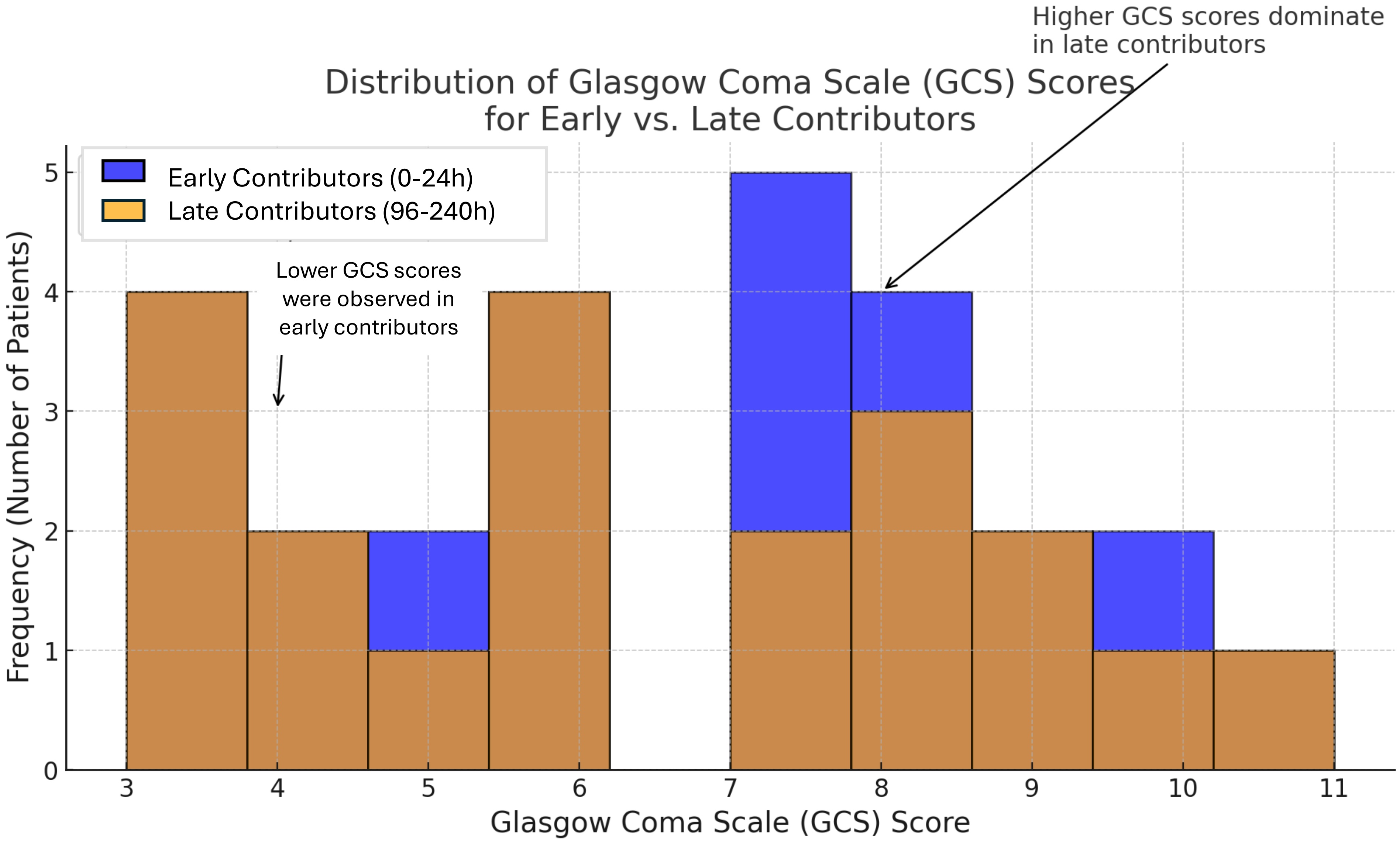

Distribution of Glasgow Coma Scale (GCS) Scores
for Early vs. Late Contributors
Higher GCS scores dominate
in late contributors
Early Contributors (0-24h)
Late Contributors (96-240h)
Lower GCS scores
were observed in
early contributors
Frequency (Number of Patients)
0
1
2
3
4
5
3
4
5
6
7
8
9
10
11
Glasgow Coma Scale (GCS) Score

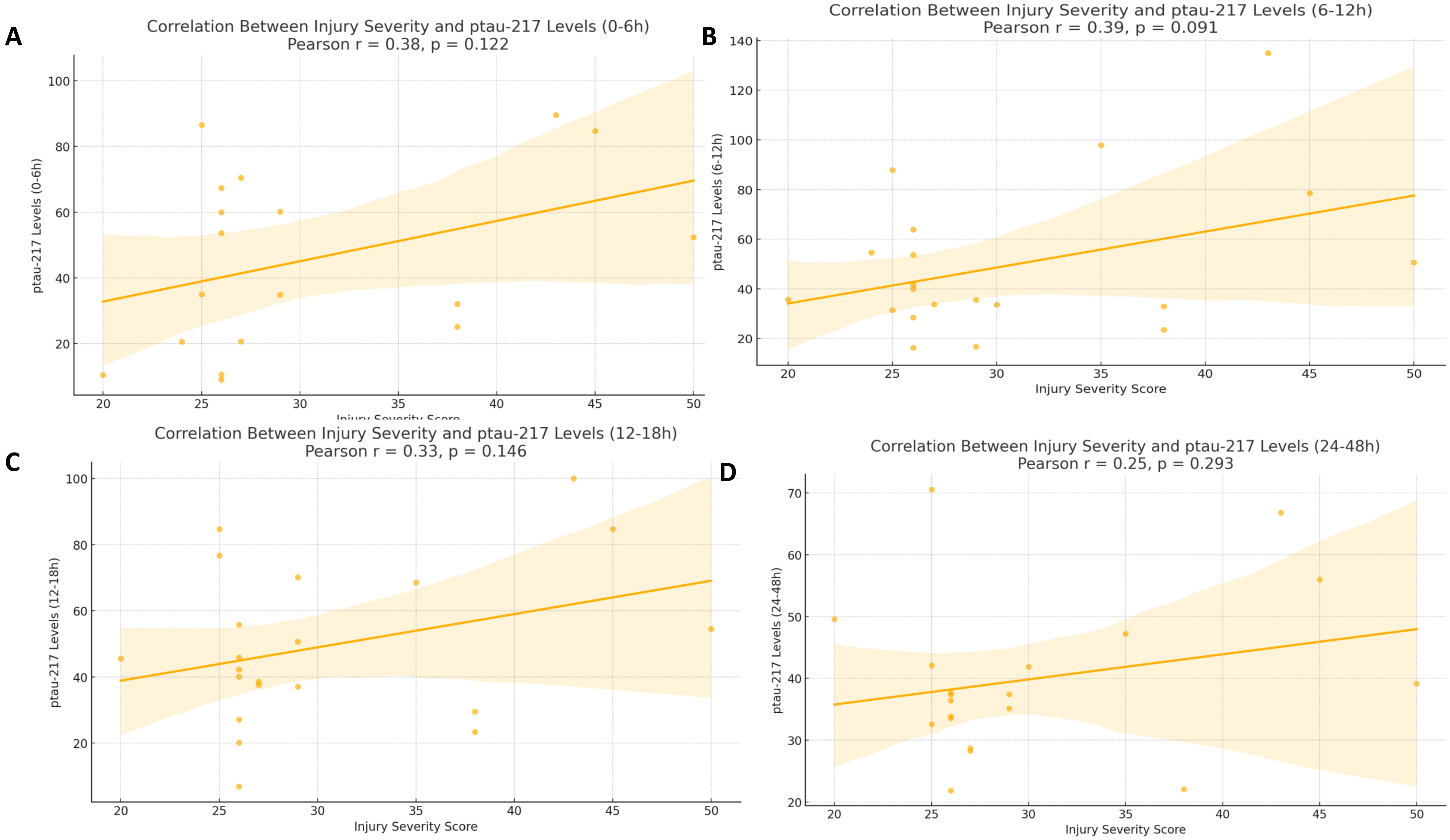

A
Correlation Between Injury Severity and ptau-217 Levels (0-6h)
Pearson r = 0.38, p = 0.122
ptau-217 Levels (0-6h)
B
Correlation Between Injury Severity and ptau-217 Levels (6-12h)
Pearson r = 0.39, p = 0.091
ptau-217 Levels (6-12h)
Injury Severity Score
C
Correlation Between Injury Severity and ptau-217 Levels (12-18h)
Pearson r = 0.33, p = 0.146
ptau-217 Levels (12-18h)
Injury Severity Score
D
Correlation Between Injury Severity and ptau-217 Levels (24-48h)
Pearson r = 0.25, p = 0.293
ptau-217 Levels (24-48h)
Injury Severity Score

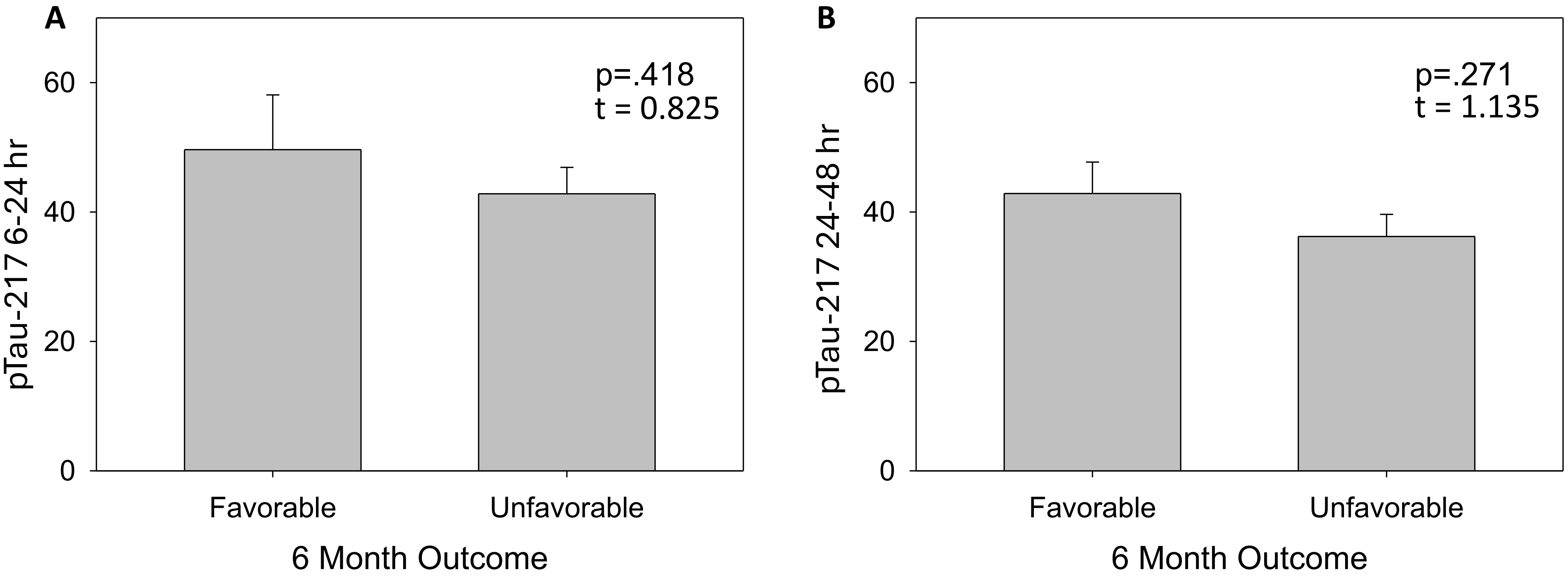
A
p=.418
t = 0.825
pTau-217 6-24 hr
0
20
40
60
Favorable
Unfavorable
6 Month Outcome
B
p=.271
t = 1.135
pTau-217 24-48 hr
0
20
40
60
Favorable
Unfavorable
6 Month Outcome
