# Supplementary material for: Early cerebrospinal fluid elevations of pTau-217 in severe traumatic brain injury subjects
Source: Front Neurol. 2025 Jul 30;16:1632679. doi: 10.3389/fneur.2025.1632679 (PMC12344560; doi:10.3389/fneur.2025.1632679)
Supplement: Supplementary file 2 [file Supplementary_file_1.docx]

# Supplementary Methods

## pTau‑217 Direct ELISA: Full Protocol and Optimization Details

The following protocol provides the complete, step-by-step methodology used to quantify phosphorylated tau at threonine-217 (pTau-217) in cerebrospinal fluid (CSF) samples from patients with severe traumatic brain injury (sTBI), adapted from a colorimetric cell-based ELISA kit (Assay Genie, CBCAB01637). The abbreviated version included in the main manuscript is summarized in Figure S1; this supplement preserves all technical details required for replication.

**pTau‑217 Direct Enzyme‑Linked Immunosorbent Assay (ELISA)**

**Analytical overview**

The performance of the pTau‑217 assay was evaluated following standard phospho‑tau assay validation procedures (**Figure S1**). A colorimetric cell‑based ELISA kit (Assay Genie, CBCAB01637), initially designed for pTau‑534/217 detection in cultured cells, was adapted for direct measurement of pTau‑217 in human CSF.

**Key antibody**

The primary antibody supplied in the kit (anti-Tau pThr217) was used for both ELISA and confirmatory Western blot (WB) to ensure epitope consistency across platforms.

**Significant modifications for CSF application**

•Sample format: Cell‑seeding steps were omitted; 100 µL aliquots of diluted CSF were added directly to high‑binding ELISA wells.

•Incubation Antigen‑coating was extended to 16h at 4°C to maximise adsorption of soluble CSF tau species.

•Standards Purified recombinant human non‑phosphorylated Tau‑441 (SignalChem-T08‑54N‑50) and dual‑kinase‑phosphorylated Tau‑441 (GSK‑3β+DYRK1A; SignalChem-T08‑50FN‑50 and T08‑50RN‑50) replaced the kit’s cell‑lysate standards.

•Specificity controls: A rabbit IgG polyclonal isotype control (Cell-Signalling: #3900) and a total‑Tau antibody (Dako-A0024) were run in parallel to verify specificity and background.

Standard curve: Protein standards were prepared at 0, 10, 30, 100, 300 pg mL⁻¹, 1, 30, 100, 300, 500 ng mL⁻¹ and 1 µg mL⁻¹ in carbonate–bicarbonate coating buffer (0.05 M, pH 9.6). Aliquots were stored at –80°C; working dilutions were made fresh.

Plate coating (Day-1): Ten microliters of neat CSF were diluted with 90 µL of coating buffer (final concentration: 10 µg total protein) and loaded into duplicate wells. Plates were sealed, shaken at 500 rpm for 30 min at room temperature, then incubated 16h at 4 °C.

**Blocking & primary antibody**

(Day-2): After four 5‑min washes with 200 µL 1% Tween‑20/TBS, wells were blocked with 200µL kit blocking buffer for 1h at RT. Plates were washed and incubated overnight at 4 C with 50µL anti‑pTau‑217 antibody (1 µg mL⁻¹) diluted in antibody diluent.

Secondary antibody (Day-3): Plates were washed four times; 50 µL HRP‑conjugated anti‑rabbit IgG (1×) were added and incubated 1.5h at RT with gentle agitation (550 rpm).

Colour development: After four washes, 100 µL ready‑to‑use Tetramethylbenzidine (TMB) substrate were added. Plates were developed for 30 min at 25°C, protected from light for a final 5 min, and read at 600 nm (pre-stop reference). The reaction was stopped with 100 µL 2 M H₂SO₄; absorbance was measured at 450 nm.

Assay performance: The 10‑point standard curve produced a third‑order polynomial fit with R²=0.843 over 0–1 µg mL⁻¹. Background (isotype) OD was <5% of the 10 pg mL⁻¹ standard. All samples were analysed in duplicate; intra‑assay coefficient of variation (CV) was 7.6%, inter‑assay CV 9.4% across three independent runs.

Western blot validation: To confirm ELISA specificity, CSF pools (control vs sTBI) and standard proteins were resolved by SDS‑PAGE, transferred to PVDF, and probed with the same anti‑pTau‑217 antibody. Bands corresponding to monomeric (~50 kDa) and oligomeric (~100 kDa) tau were quantified by densitometry, which paralleled the ELISA findings.

**Reagents and catalogue numbers**

•Anti‑Tau (pThr217) capture antibody: kit component (Assay Genie CBCAB01637)

•Recombinant Tau‑441: SignalChem T08‑54N‑50

•Dual‑kinase‑phosphorylated Tau‑441: SignalChem T08‑50FN‑50 / T08‑50RN‑50

•Isotype IgG control: Cell Signalling 3900

•Total‑Tau antibody: Dako A0024

•Protease/phosphatase inhibitors: Roche; Thermo Scientific.

Safety note:” All human CSF samples were handled under BSL‑2 conditions; institutional biosafety protocols were followed.

Troubleshooting tips: (1) Prolong coating step or increase CSF volume if OD values < background+0.05. (2) Use fresh TMB and ensure plate reader linearity at high OD values. (3) If a high background occurs, verify the Tween-20 concentration in the wash buffer and extend the wash time.
